# Glial Scar-Targeted Modification of Hepatocyte Growth Factor-Derived Peptide for Spinal Cord Injury Therapeutic Repair

**DOI:** 10.34133/bmr.0431

**Published:** 2026-10-27

**Authors:** Yuzhe Wu, Jingwei Shi, Ruize Miao, Linyi Xiang, Jiacheng Zhang, Weiyi Chi, Yuheng Zhang, Yibo Geng, Xiong Cai, Yun Shu, Jiayi Zhao, Junsheng Zhu, Xiaoqiong Jiang, Haojie Zhang, Zhenglin Li, Xiangyang Wang, Sunren Sheng, Chenchen Zhu, Jianjun Qi, Jian Xiao, Kailiang Zhou

**Affiliations:** ^1^ Department of Orthopaedics, The Second Affiliated Hospital and Yuying Children’s Hospital of Wenzhou Medical University, Wenzhou 325027, China.; ^2^ Zhejiang Provincial Key Laboratory of Orthopaedics, Wenzhou 325027, China.; ^3^ Cixi Biomedical Research Institute, Wenzhou Medical University, Ningbo 315300, China.; ^4^ The First Clinical Medical College (School of Information and Engineering), Wenzhou Medical University, Wenzhou 325035, China.; ^5^ The Second Clinical Medical College of Wenzhou Medical University, Wenzhou 325027, China.; ^6^ Department of Orthopaedics, Tangdu Hospital, The Air Force Medical University, Xi’an 710038, China.; ^7^ School of Pharmaceutical Science, Wenzhou Medical University, Wenzhou 325000, China.; ^8^ Oujiang Laboratory (Zhejiang Lab for Regenerative Medicine, Vision and Brain Health), School of Pharmaceutical Sciences, Wenzhou Medical University, Wenzhou, Zhejiang, China.; ^9^ Department of Orthopedics, Xuanwu Hospital of Capital Medical University, Beijing 100053, China.; ^10^ Department of Wound Repair, The First Affiliated Hospital of Wenzhou Medical University, Wenzhou, Zhejiang 325000, China.; ^11^ Department of Ophthalmology, The First Affiliated Hospital of Bengbu Medical University, Bengbu 233000, China.; ^12^ Department of Laboratory Medicine, The First Affiliated Hospital of Wannan Medical College (Yijishan Hospital of Wannan Medical College), Wuhu 241001, China.

## Abstract

Spinal cord injury (SCI) causes irreversible neural damage and functional impairment. H-RN is a short peptide originating from the kringle 1 domain of hepatocyte growth factor. Despite its efficacy in inhibiting inflammatory responses, the precise neuroprotective mechanisms of the peptide H-RN in SCI remain unclear. The short peptide CAQK can target the lesion epicenter of SCI. To explore the role of the peptide H-RN in SCI and address the numerous challenges faced by conventional drug therapies, this study developed a synthetic peptide, CAQK-H-RN. Our results revealed that CAQK-H-RN could cross the blood–spinal cord barrier, precisely target the SCI region, and increase drug accumulation at the injury site, thereby reducing glial scar formation, decreasing motor neuron death, and enhancing functional recovery after SCI. RNA sequencing was performed to explore the molecular mechanisms of CAQK-H-RN in SCI, which revealed that CAQK-H-RN inhibited pyroptosis and ameliorated lysosomal membrane permeabilization (LMP)-induced autophagic flux disorders in neurons after SCI. Additionally, CAQK-H-RN alleviated LMP by suppressing acid sphingomyelinase activity through the p38 mitogen-activated protein kinase/Ets-like protein-1 (Elk-1)/early growth response protein 1 (Egr-1) signaling cascade and subsequently enhanced autophagy, thereby mitigating neuronal death. This intravenous therapeutic system centered on the synthetic peptide CAQK-H-RN contributes to the functional restoration of SCI, which may be clinically applicable to spinal cord repair.

## Introduction

Spinal cord injury (SCI) is a severe and debilitating condition characterized by disruptions in sensory, motor, and autonomic neural functions. In more severe cases, it may lead to permanent paralysis, substantially decreasing life expectancy and quality of life [1]. Despite ongoing research, no consistently effective therapeutic approaches are available for treating SCI. SCI progression is typically categorized into 2 distinct phases: primary injury and secondary injury. Primary injury refers to immediate tissue damage caused by mechanical forces. Secondary injury subsequently develops and involves numerous pathological cascades, such as ischemia, oxidative stress, neuroinflammatory responses, and neuronal cell death [2]. Given the irreparable and unpredictable nature of the primary injury, contemporary treatment strategies primarily target secondary pathological events to improve patient prognosis and therapeutic outcomes. Although the complexity of the mechanisms underlying secondary SCI remains incompletely understood, current evidence strongly indicates that inhibiting neuroinflammation and neuronal death during this secondary phase is essential for SCI management [3]. Pyroptosis, a novel form of inflammatory cell death, has been identified in various tissues, including the spinal cord and brain [4]. Pyroptosis can be classified as either canonical (caspase-1 dependent) or noncanonical. During canonical pyroptosis, Toll-like receptors and NOD-like receptors (NLRs) recognize pathogen- or danger-associated molecular patterns, thereby activating inflammasome-related pathways. Mechanistically, pyroptosis is driven by inflammasome activation—particularly that of the NLR family pyrin domain containing 3 (NLRP3) inflammasome—leading to caspase-1 cleavage, which in turn matures proinflammatory cytokines (interleukin 1β [IL-1β] and interleukin 18 [IL-18]) and cleaves gasdermin D (GSDMD). The resulting N-terminal GSDMD fragments form membrane pores (10 to 15 nm), promoting cell lysis and perpetuating inflammatory cascades. In SCI, extensive signal transduction cascades markedly increase NLRP3 inflammasome expression, inducing neuronal pyroptosis and worsening secondary tissue damage [5]. Thus, inhibiting neuroinflammation and pyroptosis represents a promising therapeutic strategy.

Autophagy is a lysosome-dependent cellular degradation mechanism that eliminates impaired organelles, aggregated proteins, and other cytoplasmic substances, conferring neuroprotection in neurons and microglia in central nervous system (CNS) diseases [6]. Autophagy reduces neuronal injury and facilitates functional recovery after SCI through mechanisms such as enhancing axon regeneration, modulating the microtubule cytoskeleton, and attenuating pyroptotic cell death [7]. This catabolic pathway involves the sequential formation of phagophores, maturation into autophagosomes, fusion with lysosomes to form autolysosomes, and the subsequent degradation of internalized materials. Thus, lysosomal integrity is crucial for efficient autophagic flux [8]. Lysosomal dysfunction, particularly increased lysosomal membrane permeability (LMP), leads to the leakage of enzymes and other lysosomal components into the cytoplasmic and extracellular compartments, resulting in impaired cellular function, cell death, and disruption of the normal autophagic process [9]. Thus, the maintenance of lysosomal membrane stability is critical to ensure effective autophagic flux. Preventing LMP may restore neuronal autophagic activity, ultimately enhancing recovery from SCI. The activity of acid sphingomyelinase (ASMase), a prominent member of the sphingomyelinase enzyme family, is integral to sphingolipid metabolism [10]. ASMase predominantly localizes to the inner lysosomal membrane, where it converts sphingomyelin (SM) into ceramide. Ceramide is subsequently metabolized to sphingosine, a molecule that promotes LMP [11]. Therefore, targeting ASMase activity to reduce LMP represents a promising therapeutic approach for restoring autophagy, enhancing neuronal viability, and improving functional outcomes following SCI. Additionally, increasing evidence has revealed the crucial involvement of ASMase in SCI pathology, where ASMase activation is driven by the p38 mitogen-activated protein kinase (MAPK) signaling pathway through the Ets-like protein-1 (Elk-1)/early growth response protein 1 (Egr-1) transcriptional axis. Specifically, after SCI, activated p38 MAPK translocates into the nucleus, phosphorylates Elk-1, and facilitates its interaction with CREB-binding protein and serum response factor to form a transcription complex. This complex subsequently enhances the transcription of Egr-1, a key nuclear transcription factor. Egr-1 directly interacts with the EGR sequence within the promoter region of the *Smpd1* gene, thus up-regulating ASMase expression [12].

Hepatocyte growth factor (HGF), a multifunctional cytokine, is structurally characterized as a disulfide-linked α/β heterodimer with a molecular weight of approximately 90 kDa. The α-chain comprises an N-terminal region and 4 kringle domains (K1 to K4). Notably, the high-affinity binding site for the MET receptor is formed primarily by the K1 domain in conjunction with the N-terminal region, with key residues located within the K1 domain [13]. In addition to its well-known hepatocyte mitogenic activity, HGF, as a multifunctional protein, plays protective roles in inflammation modulation, tissue regeneration, and wound healing processes after injury. Moreover, HGF is recognized as a potent neurotrophic and angiogenic molecule within the CNS that facilitates endogenous regenerative responses and functional recovery following SCI, highlighting its therapeutic potential [14]. Nevertheless, high-molecular-weight proteins such as HGF encounter significant challenges for clinical translation in neurological conditions because of factors such as rapid systemic clearance, limited permeability across the blood–brain barrier, and inadequate delivery systems. Consequently, short peptides derived from key active components of certain functional proteins have garnered attention because of their promising potential in targeting neurological disease [15]. Among these peptides, H-RN, a novel peptide (sequence: RNPRGEEGGPW; molecular weight: 1,254.34 Da) originating from the kringle 1 domain of HGF, exerts anti-inflammatory effects comparable to those of the parent protein [16]. Moreover, previous studies have demonstrated that peptide-based therapeutics markedly ameliorate SCI outcomes by suppressing pyroptosis and LMP [17]. These findings suggest potential therapeutic benefits of H-RN in SCI; however, its detailed functional role remains to be elucidated.

Currently, methylprednisolone remains the sole clinically approved treatment for mitigating acute inflammatory responses in patients with SCI. Although numerous pharmacological agents have been explored to target neuronal injury and microglial inflammation during the secondary injury phase, their clinical application remains challenging. Limitations include short retention times at the lesion site, poor accumulation, the absence of controlled-release mechanisms, and low drug delivery efficiency due to the blood–spinal cord barrier [18,19]. Furthermore, inadequate targeting of specific cell populations in the injury microenvironment compromises neuroprotective effects [20]. Although intravenous administration remains clinically common, the effective drug concentration reaching the SCI site is extremely low. Consequently, enhancing the spinal-cord-targeting ability of intravenously delivered drugs is critically important. Peptide-based targeting has emerged as a promising therapeutic approach for multiple diseases, with small peptides often possessing the ability to penetrate the blood–brain barrier, thereby offering a potential advantage for CNS targeting [21–23]. The peptide CAQK (cationic cysteine–alanine–glutamine–lysine) displays strong affinity and specificity for proteoglycan-rich regions at the SCI lesion epicenter [24]. In the present study, we synthesized a peptide, CAQK-H-RN, for the first time with the aim of enhancing selective targeting of the injury site. Previous research has indicated that HGF effectively suppresses pyroptotic pathways and facilitates neuronal repair following acute ischemic stroke [25]. Nevertheless, whether H-RN alone or in combination with CAQK (CAQK-H-RN) inhibits ASMase-dependent LMP and consequently restores autophagic function and prevents pyroptosis after SCI remains unclear. Therefore, this investigation assessed the therapeutic efficacy of CAQK-H-RN in an SCI mouse model. Specifically, our primary objectives were to determine (a) whether CAQK-H-RN selectively targets lesion sites of SCI, promoting neuronal protection and the recovery of motor function; (b) the involvement of autophagy, LMP, and pyroptosis in mediating the neuroprotective effects of CAQK-H-RN; and (c) whether ASMase is critically involved in the beneficial outcomes induced by CAQK-H-RN treatment, particularly through the modulation of the p38 MAPK signaling pathway.

## Materials and Methods

### Peptide synthesis

The peptide H-RN (RNPRGEEGGPW) and the synthetic peptide CAQK-H-RN (CAQK-RNPRGEEGGPW) were synthesized by Wuhan Tiande Biotechnology Co., Ltd. (Hubei, China) using the high-efficiency solid-phase method. The basic principle involves first covalently attaching the carboxyl groups of the C-terminal amino acids of the desired peptide chain to an insoluble high-molecular-weight resin. Afterward, the amino groups of these bound amino acids are deprotected and undergo a condensation reaction with the carboxyl groups of the next amino acid in the presence of a condensing agent, forming amide bonds and extending the peptide chain. This process is repeated (condensation → washing → deprotection → neutralization and washing → next round of condensation) until the desired length of the peptide chain is achieved. Finally, the peptide chain is cleaved from the resin and, after purification and freeze-drying, the desired peptide is obtained. The purity of the final product was greater than 98% and was characterized by analytical high-performance liquid chromatography (HPLC) and mass spectrometry. All peptides were stored at −20 °C and dissolved in dimethyl sulfoxide before the experiments.

### Animals

Our study exclusively examined female mice because manual urination is more feasible for preventing urinary retention after SCI due to the relatively shorter urethral length in female animals. Therefore, female animals are frequently used in experimental SCI research. For this study, healthy adult female C57BL/6J mice (6 to 8 weeks old, average body weight of 20 to 25 g) were acquired from the Experimental Animal Center of our university (Zhejiang, People’s Republic of China), with the institutional certification number SCXK (ZJ) 2015-0001. All mice were housed under standard conditions (21 to 25 °C, 12-h light/dark cycle, 50% to 60% humidity) with free access to food and water.

### Reagents and antibodies

The peptide H-RN (HPLC ≥ 98%, cat. no. TD-M10-012) and the synthetic peptide CAQK-H-RN (HPLC ≥ 98%, cat. no. TD-M10-013) were purchased from Wuhan Tiande Biotechnology Co., Ltd. (Hubei, China). Pentobarbital sodium, Masson staining reagent, and hematoxylin–eosin (HE) staining reagent were obtained from Solarbio Science & Technology in Beijing, China. Chloroquine (CQ; cat. no. C6628), asiatic acid (AA; cat. no. A2612), a protease inhibitor cocktail (cat. no. P8340), and phosphatase inhibitor cocktail III (cat. no. P0044) were supplied by Sigma-Aldrich (St. Louis, MO, USA). A lysosome enrichment kit for tissue and cultured cells (cat. No. 89839) was obtained from Thermo Fisher Scientific (Waltham, USA). Primary antibodies against Beclin-1 (cat. no. 3738), LC3B (cat. no. 3868), NLRP3 (cat. no. 15101), p38 (cat. no. 8690t), and p-p38 (cat. no. 4511t) were obtained from Cell Signaling Technology (Beverly, MA, USA). Antibodies against vacuolar protein sorting 34 (VPS34; cat. no. 12452-1), cathepsin D (CTSD; cat. no. 21327-1), caspase-1 (cat. no. 22915-1), and glyceraldehyde-3-phosphate dehydrogenase (GAPDH; cat. no. 104941) were provided by The Proteintech Group (Chicago, IL, USA). Goat anti-mouse IgG H&L Alexa Fluor 594 (cat. no. ab150116), Alexa Fluor 488 (cat. no. ab150113), goat anti-rabbit IgG H&L Alexa Fluor 488 (cat. no. ab150077), Alexa Fluor 647 (cat. no. ab150079), Alexa Fluor 594 (cat. no. ab150080), anti-neurofilament heavy polypeptide (cat. no. ab207176), chondroitin sulfate proteoglycan (CSPG; CS-56) (cat. no. ab11570), apoptosis-associated speck-like protein containing a caspase recruitment segment (ASC; cat. no. ab180799), Egr-1 (cat. no. ab300449), synaptophysin (SYN; cat. no. 32127), microtubule-associated protein-2 (MAP2; cat. no. ab5392), mouse monoclonal antibodies against NeuN (cat. no. ab104224), and rabbit monoclonal antibodies against NeuN (cat. no. ab177487), p62/sequestosome 1 (p62/SQSTM1; cat. no. ab240635), cathepsin B (CTSB; cat. no. ab214428), and lysosome-associated membrane protein 1 (LAMP1; cat. no. ab24170) were purchased from Abcam (Cambridge, UK). The cathepsin L (CTSL) antibody (cat. no. AF1515) was provided by R&D Systems (Minnesota, USA). Elk-1 (cat. no. A0789), IL-1β (cat. no. A1112), and IL-18 (cat. no. A1115) antibodies were provided by ABclonal Technology (Cambridge, MA, USA). NLR family pyrin domain containing 1 (NLRP1) (cat. no. DF13187), p-Elk-1 (cat. no. AF3212), ASMase (cat. no. DF13384), GSDMD-N (cat. no. DF12275), extracellular signal-regulated kinase 1/2 (ERK1/2; cat. no. AF0155), p-ERK1/2 (cat. no. AF1015), c-Jun N-terminal kinase (JNK; cat. no. AF6318), and p-JNK (cat. no. AF3318) antibodies were obtained from Affinity Biosciences (Ohio, USA). Anti-glial fibrillary acidic protein (anti-GFAP; cat. no. sc-166458) antibodies were provided by Santa Cruz Biotechnology (Dallas, TX, USA). 4′,6-Diamidino-2-phenylindole (DAPI) solution was obtained from Beyotime Biotechnology (Jiangsu, China). A bicinchoninic acid (BCA) kit (cat. no. 23227) and NE-PER nuclear and cytoplasm extraction reagents (cat. no. 78835) were acquired from Thermo Fisher Scientific (Rockford, IL, USA).

### Animal model of SCI

Each mouse was anesthetized prior to surgery through the intraperitoneal administration of pentobarbital sodium (50 mg/kg, 1% w/v). After anesthesia, a laminectomy was carefully performed at spinal segments T9 to T10, exposing the dorsal spinal cord without damaging the dura mater. A standardized SCI was subsequently induced using a spinal impactor (W.M. Keck+, USA) by releasing a 5-g weight onto the exposed spinal surface from a height of 3 cm. Immediately after injury induction, the muscle and skin layers were sutured sequentially with 4–0 silk. Sham-operated mice underwent an identical surgical procedure, excluding the impact injury. Manual bladder evacuation was conducted 3 times daily after surgery until spontaneous bladder function was restored.

### AAV vector packaging

ASMase adeno-associated virus (AAV-ASMase) and the corresponding AAV-empty control virus were engineered and packaged by Genecast Biotechnology (Shanghai, China). Both vectors were based on AAV serotype 9 and driven by a cytomegalovirus promoter. The detailed protocols were conducted as described previously [17]. The viral titers of AAV-ASMase and AAV-empty were measured by quantitative polymerase chain reaction (qPCR). The final concentrations were determined to be 2.93 × 10^13^ viral genomes (vg)/ml for AAV-ASMase and 3.0 × 10^13^ vg/ml for the AAV-empty control.

### AAV vector injection

Previous studies have indicated that AAV vectors require several days to effectively express target genes in vivo; thus, a 2-week interval before SCI was considered optimal for vector administration. Accordingly, the mice in the SCI+AAV-ASMase and SCI+C-H-RN+AAV-ASMase groups received intraspinal injections (2 μl of viral suspension in phosphate-buffered saline [PBS]) at a depth of 1.5 mm below the dorsal spinal surface at spinal levels T9 to T10 using a micropipette. Control mice (in the SCI+AAV-Blank and SCI+C-H-RN+AAV-Blank peptide groups) received equivalent volumes of nonfunctional viral vector solutions. The micropipette needle remained in position for 1 min after the injection to prevent leakage of the vector solution. The surgical wounds were subsequently closed, and the mice were transferred to heated recovery cages. No significant hind-limb paralysis or apparent motor impairments were observed during postoperative recovery.

### Evaluation of functional behavior

Motor function recovery was evaluated using the Basso Mouse Scale (BMS) at 0, 1, 3, 7, 14, 21, and 28 d following SCI, where scores ranged from 0 (complete paralysis) to 9 (normal locomotion). Assessments were conducted in an open-field arena. Additionally, footprint analyses were performed 28 d after injury, with the forepaws and hindpaws marked with blue and red dyes, respectively. The hind-limb stride length was quantified by measuring the distances between consecutive hindpaw footprints, and the extent of toe dragging was determined by calculating the ratio of the hind-limb drag distance to the total movement distance. All behavioral testing was conducted independently by 2 blinded observers. Comprehensive hind-limb movement assessments were conducted based on established methodologies [26]. Following protocol optimization, the mice were subjected to straight-line locomotion tests on a runway. Movement trajectories were captured using a high-speed camera and subsequently analyzed via the DeepLabCut GUI 2.3.4 software, with anatomical landmarks identified at the iliac crest, hip, knee, and ankle joints and toes. The MATLAB 2022a software was then employed to generate hind-limb stick-figure representations and calculate joint angles at the hip, knee, and ankle. The swing and stance phases are illustrated in red and black, respectively. Radar charts were constructed to depict amplitude variations in height and joint angles for the sham, SCI, SCI+H-RN, and SCI+C-H-RN groups. The maximum joint elevations (hip, knee, and ankle) and reaction times are further summarized in bar charts.

### Groups

Except for the RNA sequencing experiments (biological replicates *n* = 3), all groups included 5 biological replicates (*n* = 5). In total, 140 C57BL/6J mice were randomized into 12 experimental conditions: sham (*n* = 15), SCI (*n* = 15), SCI+H-RN (*n* = 15), SCI+C-H-RN (*n* = 30), SCI+CQ (*n* = 5), SCI+C-H-RN+CQ (*n* = 10), SCI+AA (*n* = 5), SCI+C-H-RN+AA (*n* = 5), SCI+AAV-Blank (*n* = 10), SCI+AAV-ASMase (*n* = 10), SCI+C-H-RN+AAV-Blank (*n* = 10), and SCI+C-H-RN+AAV-ASMase (*n* = 10). The mice in the SCI+H-RN group received an intraperitoneal injection of the peptide H-RN (300 μg/kg) 30 min prior to the surgical procedures. Animals in the SCI+C-H-RN, SCI+C-H-RN+AA, SCI+C-H-RN+CQ, SCI+C-H-RN+AAV-Blank, and SCI+C-H-RN+AAV-ASMase groups were similarly administered the synthetic peptide CAQK-H-RN (300 μg/kg) intraperitoneally 30 min before surgery, followed by daily injections for 3 d postoperatively. The dosing regimen and timing for both the H-RN peptide and the synthetic peptide CAQK-H-RN were selected based on previously documented studies and dose–response evaluations. The control groups, including the SCI, SCI+AAV-Blank, sham, and SCI+AAV-ASMase groups, received injections of identical volumes without active peptides. Additionally, CQ (60 mg/kg) or AA (550 μg/kg) was administered intraperitoneally 30 min prior to the peptide injection, which was repeated daily for 3 consecutive days, as recommended by earlier CNS injury studies [27]. At designated time points (3 and 28 d) following surgery, the mice were euthanized using an overdose of pentobarbital sodium, after which spinal cord samples were collected for subsequent histological examination.

### Tissue pretreatment for HE and Masson’s trichrome staining

At 28 d after surgery, the mice were deeply anesthetized and euthanized through cardiac perfusion with ice-cold PBS (100 mM, pH 7.4) followed by fixation with a 4% paraformaldehyde solution. Spinal cord tissues (10-mm segments centered on the lesion epicenter) were carefully dissected and postfixed for 24 h with 4% paraformaldehyde. Following fixation, the spinal specimens were embedded in paraffin wax and sectioned longitudinally. For HE staining, tissue sections (5 μm thick) were prepared using a microtome and mounted onto slides coated with poly-l-lysine. For Masson’s trichrome staining, longitudinal sections were first deparaffinized and treated with mordant solution (10% potassium dichromate mixed with 10% trichloroacetic acid) and then counterstained with hematoxylin. Differentiation was subsequently performed in ethanol–hydrochloric acid, and the slides were briefly immersed in dilute ammonia solution for bluing, after which Masson’s trichrome staining solution was applied. Images were obtained using an optical microscope (Olympus, Tokyo, Japan).

### Western blotting

The mice were humanely euthanized on day 3 postinjury, and spinal cord tissues (1-cm segments) centered on the lesion site were carefully excised and homogenized in ice-cold radioimmunoprecipitation assay lysis buffer containing protease and phosphatase inhibitors. Protein extraction was performed using specialized reagents, and protein concentrations were measured in a BCA assay. Equal amounts of protein (60 μg) were subsequently subjected to electrophoretic separation on 12% sodium dodecyl sulfate–polyacrylamide gel electrophoresis gels, followed by transfer to polyvinylidene fluoride membranes (Millipore). The membranes were blocked with 5% skim milk for 2 h at room temperature and then incubated overnight at 4 °C with primary antibodies (1:1,000 dilution) against IL-1β, GSDMD-N, IL-18, Beclin-1, caspase-1, ASC, CTSL, CTSB, CTSD, NLRP1, NLRP3, SQSTM1/p62, microtubule-associated protein 1 light chain 3 (LC3), VPS34, LAMP1, GAPDH, ASMase, ERK1/2, p-ERK1/2, p38, p-p38, JNK, p-JNK, Elk-1, p-Elk-1, and Egr-1. Following the washing steps, the membranes were incubated with horseradish peroxidase-conjugated secondary antibodies at room temperature for 2 h. The protein bands were visualized using an enhanced chemiluminescence detection system and the Bio-Rad ChemiDoc XRS+ Imaging System.

### Immunofluorescence staining

Spinal cord segments (transverse section: 10-mm length, 4 mm rostral to the lesion epicenter; longitudinal section: 10-mm length centered on the lesion epicenter) were harvested at 1, 3, and 28 d post-SCI to prepare paraffin sections. The paraffin sections were deparaffinized in xylene, rehydrated through a graded ethanol series, and subjected to antigen retrieval in 10 mM sodium citrate buffer (pH 6.0) at 95 °C for 20 min. The sections were permeabilized with 0.5% Triton X-100 in PBS (10 min), blocked with 10% bovine serum albumin/PBS (1 h at room temperature), and incubated overnight at 4 °C with primary antibodies against (transverse section: caspase-1, GSDMD-N, SQSTM1/p62, MAP2, SYN, NeuN, LC3, ASMase, and CTSB; longitudinal section: GFAP and neurofilament heavy polypeptide [NF-H]). After being washed with PBS (3 × 10 min), the sections were incubated with Alexa Fluor-conjugated secondary antibodies (1:1,000) for 1 h at 37 °C and counterstained with DAPI (1 μg/ml) for 10 min. Images of transverse sections, located 3 mm rostral to the lesion, were captured using an Olympus IX83 inverted microscope (Olympus, Tokyo, Japan). Images of longitudinal sections, 10 mm in length and centered on the lesion epicenter, were captured using an Olympus IX83 inverted microscope (Olympus, Tokyo, Japan). Five randomly selected microscopic fields from the anterior horn region of each section were analyzed using the ImageJ software to quantify the integrated fluorescence intensities of MAP2, caspase-1, GSDMD-N, SQSTM1/p62, GFAP, CSPG, NF-H, and ASMase. Moreover, LC3 II-positive puncta, indicative of autophagosomes (0.2 to 10 μm in size), were counted in each neuron via ImageJ analysis. SYN-positive synapses on motor neurons were quantified manually by 2 independent observers who were blinded to the group assignments, ensuring unbiased data collection.

### Subcellular partitioning and isolation of the lysosome-rich fraction

Spinal cord tissue fragments (5 mm in length) were subjected to ice-cold homogenization using a Dounce tissue grinder. The lysosomal fraction was isolated from the tissue homogenate via differential centrifugation using the Lysosome Enrichment Kit for Tissue and Cultured Cells (Thermo Fisher Scientific, 89839) according to the manufacturer’s instructions. The resulting supernatant fractions were carefully preserved as the cytosolic fraction.

### Enzyme-linked immunosorbent assay

The enzymatic activities of ASMase (Bioswamp, MU32986), CTSB (Bioswamp, MU31270), and α-*N*-acetylglucosaminidase (NAGLU; Bioswamp, MU30875) were assessed using enzyme-linked immunosorbent assay (ELISA) kits in accordance with the manufacturer’s protocols. ASMase, CTSB, and NAGLU were quantified by measuring the optical densities of the samples at 550 nm using a microplate reader. To ensure accuracy, a correction wavelength of 450 nm was used during the quantification process.

### Quantitative PCR

Total RNA was extracted from the spinal cord using TRIzol reagent according to the manufacturer’s instructions. Quantitative analysis was performed through a 2-step reaction procedure: reverse transcription (RT) and PCR. Each RT reaction contained 0.5 μg of RNA, 2 μl of 5× TransScript All-in-One SuperMix for qPCR, and 0.5 μl of genomic DNA remover, with a total reaction volume of 10 μl. The reaction was performed using GeneAmp PCR System 9700 (Applied Biological Systems, USA) at 42 °C for 15 min, followed by 5 s at 85 °C. The 10-μl RT reaction mixture was then diluted 10-fold in nuclease-free water and incubated at −20 °C. Real-time PCR was performed using the LightCycler 480 II Real-Time PCR System (Roche, Switzerland) with a 10-μl PCR mixture containing 1 μl of complementary DNA, 5 μl of 2× PerfectStart Green qPCR SuperMix, 0.2 μl of the forward primer, 0.2 μl of the reverse primer, and 3.6 μl of nuclease-free water. The samples were incubated in a 384-well optical plate (Roche) at 94 °C for 0.5 min, followed by 45 cycles at 94 °C for 5 s and 60 °C for 30 s. All samples were analyzed 3 times. After the PCR cycles, melting curve analyses were performed to verify the specific generation of the anticipated PCR products. The following primer sequences were developed in the laboratory and synthesized by Shanghai Generay Biotechnology based on the messenger RNA (mRNA) sequences acquired from the National Center for Biotechnology database: Smpd1, 5′-AGGGGAGGAGCTGTTTTGTG-3′ (forward) and 5′-CATTGCCAGGCTCGTAGACA-3′ (reverse). Finally, the mRNA expression levels were normalized to those of *β-actin* and computed via the ^ΔΔ^CT approach.

### RNA sequencing and functional enrichment analysis

Spinal cord tissue samples encompassing the injured segment at the lesion site were harvested 3 d after SCI, and total RNA was extracted using TRIzol reagent according to the manufacturer’s protocol. The RNA quality was assessed by determining the concentration and purity with a NanoDrop 2000 spectrophotometer (Thermo Scientific, USA), and RNA integrity was evaluated with Agilent 2100 Bioanalyzer (Agilent Technologies, USA). RNA libraries were subsequently generated using the TruSeq Stranded mRNA LT Sample Prep Kit (Illumina, San Diego, CA, USA) according to standard instructions. OE Biotech Co., Ltd. (Shanghai, China) performed the transcriptome sequencing and subsequent bioinformatics analyses. The Illumina HiSeq X Ten system was used for sequencing, yielding paired-end reads (125 or 150 bp). The raw sequencing data were subjected to quality filtering using Trimmomatic to eliminate low-quality sequences and poly-N-containing reads, thereby generating high-quality reads for downstream analysis. Clean reads were aligned to the mouse reference genome (GRCm38.p6) using the HISAT2 software. Transcript abundance was quantified as fragments per kilobase million (FPKM) values with Cufflinks, whereas gene-specific read counts were calculated using HTSeq-count. Differentially expressed genes (DEGs) were identified with the DESeq (2012) package in R, applying thresholds of *P < 0.05 and fold-change criteria (>2 or <0.5). Hierarchical clustering was conducted to identify DEG expression profiles across groups and samples. Gene Ontology (GO) analysis based on the hypergeometric distribution was also performed using the R package clusterProfiler (v4.6.0) to assess the functional enrichment of the DEGs identified.

### Single-nucleus RNA sequencing analysis

Single-nucleus RNA sequencing data were retrieved from the Gene Expression Omnibus (GEO) database (accession number: GSE172167). This dataset included lumbar spinal cord samples from female C57BL/6 mice (aged 12 to 30 weeks) collected under 5 different conditions—uninjured controls and at 1 d, 1 week, 3 weeks, and 6 weeks following injury—each with 3 biological replicates (total *n* = 15). The Harmony algorithm integrated within the Seurat package (R platform) was employed to minimize batch effects across samples. Quality control filtering involved selecting nuclei expressing at least 200 genes and exhibiting mitochondrial gene expression less than 20%. Neuronal nuclei were required to have at least 500 detectable genes per nucleus, which is consistent with the observation that neurons typically demonstrate higher gene counts than other cell types do. Genes expressed in fewer than 3 nuclei were excluded from further analyses. The top 3,000 variable genes were subsequently identified using the “FindVariableFeatures” function. Principal component analysis was performed for dimensionality reduction, and the top 30 principal components were utilized for clustering via Seurat’s “FindNeighbors” and “FindClusters” functions. Cell and cluster visualization was conducted using uniform manifold approximation and projection. Clusters with fewer than 3 distinctive marker genes, insufficient unique molecular identifier counts, or ambiguous gene expression signatures were regarded as low quality and thus excluded based on the criteria defined in prior studies. Gene expression was visually represented in violin plots and scatterplots generated with Seurat’s “VlnPlot()” and “FeaturePlot()” functions, respectively.

### In vivo imaging

In vivo imaging was performed with Cyanine5.5 (Cy5.5)-labeled H-RN and C-H-RN. At 6 h after tail vein administration, the mice were imaged with an in vivo imaging system (IVIS) (PerkinElmer, Waltham, MA, USA) to assess the in vivo fluorescence signal distribution. The mice were then sacrificed by cervical dislocation, and the heart, liver, spleen, lungs, kidneys, and spinal cord were removed for fluorescence imaging. The Cy5.5-related fluorescence signals were analyzed in the Living Image software (PerkinElmer).

### In vivo toxicity evaluation

After SCI, groups of mice were treated with PBS or the synthetic peptide CAQK-H-RN (100, 200, 300, or 400 μg/kg), and the blood from each mouse was isolated for serum biochemical assays. Major organs, including the heart, liver, spleen, lungs, and kidneys, were used for HE staining.

### Dihydroethidium staining

Spinal cord tissue was subjected to gradient dehydration in 15%, 20%, and 30% sucrose solutions. The dehydrated tissue was placed on clean filter paper to remove excess moisture, covered with the optimal cutting temperature (OCT) compound, and cooled until the OCT was completely solidified. The tissue was subsequently cut into 5-μm-thick frozen spinal cord sections. Frozen spinal cord sections were stained with dihydroethidium (DHE; Beyotime, S0063) according to the manufacturer’s protocol. Fluorescence microscopy imaging was performed using state-of-the-art equipment (Olympus, Japan) to capture high-resolution images of transverse sections positioned 3 mm rostral to the lesion site. Five random areas in the anterior horn of each sample were selected for image analysis. Quantitative assessments were performed using the ImageJ software to calculate the integrated optical density of DHE.

### Statistical analysis

Statistical analysis was performed using the GraphPad Prism software (version 10.1.2). The data are expressed as mean ± standard error of the mean, and the measured parameters were subjected to rigorous statistical analysis. In this study, a 2-tailed unpaired *t* test was employed to compare independent datasets between 2 groups. When the data were normally distributed, differences among 3 or more groups were analyzed using 2-way analysis of variance and Tukey’s multiple comparisons test. For datasets violating normality assumptions, the nonparametric Mann–Whitney *U* test was applied. Statistical significance was defined as *P < 0.05.

## Results

### Ability of the synthetic peptide CAQK-H-RN to target the injured spinal cord

Mice were treated with various peptide concentrations to determine the optimal dosage of the synthetic peptide CAQK-H-RN for facilitating motor function recovery after SCI. The results showed that 300 μg/kg was the most effective dosage (Fig. S1A). The biosafety and compatibility of CAQK-H-RN were subsequently assessed to establish its potential suitability for clinical translation. Histological analysis of primary organs (heart, liver, lung, spleen, and kidney) at 24 h after peptide injection revealed no detectable morphological abnormalities (Fig. S1B to F). Furthermore, CAQK-H-RN administration did not significantly affect body weight, liver enzyme parameters (alanine aminotransferase and aspartate aminotransferase), or renal function markers (creatinine), underscoring its favorable safety profile. Multitarget binding analyses were performed to confirm the specificity of CAQK-H-RN in targeting SCI lesions. SCI model mice received intravenous injections (tail vein) of Cy5.5-labeled peptides (C-H-RN or unmodified H-RN) at 72 h postinjury, and the fluorescence-based biodistribution was monitored using IVIS imaging. Increased fluorescence accumulation was prominently detected at lesion sites 6 h after C-H-RN administration, in contrast to the significantly weaker signals observed with H-RN alone (Fig. 1A and B). A subsequent quantitative assessment of the fluorescence in the dissected spinal cord tissue confirmed that the fluorescence intensities at the injury sites were substantially greater in the C-H-RN group than in the unmodified H-RN group (Fig. 1C and D). These data collectively demonstrated that the improvement in lesion-targeting capacity was mediated by the CAQK modification. Individual organs were dissected and analyzed by IVIS imaging to characterize the biodistribution further. Although unmodified H-RN was predominantly localized to the liver, treatment with C-H-RN notably reduced the fluorescence signals within the liver, spleen, and kidney tissues, indicating increased specificity toward the injured spinal cord (Fig. 1E and F).

**Fig. 1.**
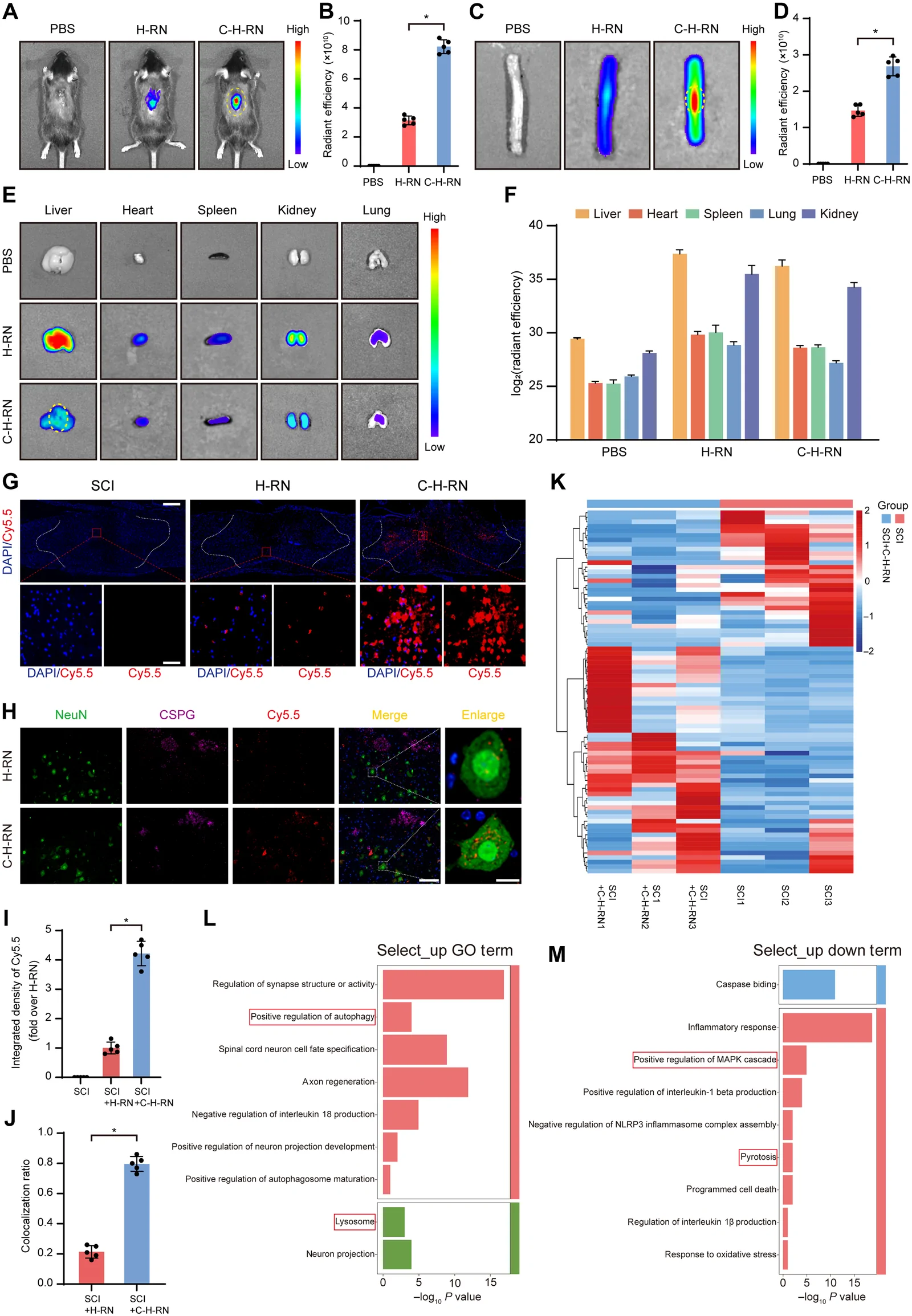
Ability of the synthetic peptide CAQK-H-RN to target the injured spinal cord. (A) Representative IVIS images of C57BL/6 mice after intravenous injection of phosphate-buffered saline (PBS), Cyanine5.5 (Cy5.5)-labeled H-RN, or Cy5.5-labeled CAQK-H-RN (color gradient: radiant efficiency). (B) Quantification of the radiant efficiency in the target regions of mice in the PBS, H-RN, and C-H-RN groups after injection. (C) Representative IVIS images of mouse spinal cords treated with PBS, Cy5.5-labeled H-RN, or Cy5.5-labeled CAQK-H-RN. (D) Quantification of the radiant efficiency in the spinal cord injury (SCI) areas ex vivo in the PBS, H-RN, and C-H-RN groups. (E) Representative IVIS images of anatomical organs in mice treated with PBS, Cy5.5-labeled H-RN, or Cy5.5-labeled CAQK-H-RN. (F) Quantification of the log_2_(radiance efficiency) of the dissected organs in the PBS, H-RN, and C-H-RN groups. (G) Representative longitudinal images of spinal cord tissue in the SCI, H-RN, and C-H-RN groups (scale bar = 1,000 μm). Local magnification shows the distribution of Cy5.5-positive cells in the injury area (scale bar = 40 μm). (H and J) Analysis of the colocalization of Cy5.5-labeled H-RN and Cy5.5-labeled C-H-RN with neurons at the center of spinal cord lesions (scale bar = 1,000 μm). Enlarged immunofluorescence images are shown (scale bar = 40 μm). (I) Quantitative analysis of the relative integrated fluorescence density of Cy5.5 in damaged areas. (K) Heatmap analysis of up-regulated and down-regulated genes induced by the administration of the synthetic peptide CAQK-H-RN to mice for 3 d (*n* = 3 mice per group). (L) Gene Ontology (GO) enrichment analysis of the up-regulated target genes to identify the biological processes influenced by synthetic peptide CAQK-H-RN treatment. (M) GO enrichment analysis of the down-regulated target genes to identify the biological processes influenced by synthetic peptide CAQK-H-RN treatment. The data are presented as mean ± standard error of the mean (SEM) (*n* = 5 mice per group); *P < 0.05 indicates significant differences; ns, not significant. For the data in (B), (D), (I), and (J), statistical analysis was performed using 2-way analysis of variance (ANOVA) followed by Tukey’s multiple comparisons test. For the data in (K) to (M), statistical analysis was performed using an unpaired *t* test.

Additional histological verification involved immunofluorescence (IF) staining of longitudinally sectioned spinal cord tissues containing the lesion area. No Cy5.5-associated fluorescence was evident in the PBS controls; however, markedly higher Cy5.5 fluorescence intensities were observed within the lesions of the C-H-RN-treated group than in those of the H-RN-treated animals, which was consistent with the IVIS imaging results (Fig. 1G and I). In addition, we explored the localization and cell-type-specific expression of Cy5.5 in the spinal cord. IF staining combined with Pearson correlation coefficient analysis revealed that 3 d after SCI, Cy5.5 was primarily colocalized with neurons (NeuN^+^) in the SCI lesion region, with much less colocalization observed in astrocytes (GFAP^+^) and microglia (Iba1^+^) (Fig. S2A to E). The colocalization of Cy5.5 fluorescence with the neuronal marker NeuN and CSPG was examined to explore the mechanisms underlying lesion targeting. Colocalization was minimal for H-RN-treated mice but was significantly increased in samples from the C-H-RN group (Fig. 1H and J). These findings imply that C-H-RN preferentially accumulates in injured regions because it binds CSPG molecules and exhibits substantial affinity for neurons.

Transcriptomic profiling was conducted on spinal cord samples from the SCI and SCI+C-H-RN groups to elucidate the underlying therapeutic mechanisms of CAQK-H-RN in SCI. Differential gene expression analysis identified 381 significantly altered genes (106 up-regulated and 275 down-regulated genes; *P < 0.05; Fig. 1K). GO functional enrichment analysis highlighted the significant involvement of biological processes related to lysosomal activity, autophagy, pyroptosis, and MAPK signaling pathways (Fig. 1L and M). Collectively, these results suggest that the therapeutic efficacy of CAQK-H-RN in SCI treatment may be mediated by the modulation of pyroptosis, autophagic activity, and the MAPK signaling pathway.

### CAQK-H-RN promotes functional recovery after SCI

We performed histological analyses and evaluated motor function outcomes to assess the neuroprotective properties of the synthetic peptide CAQK-H-RN in a mouse model of spinal cord contusion. Effective therapeutic interventions for SCI rely heavily on axonal regeneration and preservation. Therefore, IF staining with NF-H and GFAP antibodies was initially conducted to visualize axons and glial scar formation at the lesion boundary. Compared with SCI alone and H-RN peptide-treated animals, those administered CAQK-H-RN presented significantly elevated NF-H fluorescence intensity and markedly diminished GFAP immunoreactivity near the lesion site, indicating enhanced axon regeneration and reduced glial scarring (Fig. 2A to C). Additionally, the expression of MAP2, a neuronal cytoskeletal protein whose levels decrease following axonal injury, and SYN, which is indicative of synaptic plasticity and connectivity postinjury, was examined. At 28 d postinjury (dpi), IF staining revealed notable decreases in anterior horn motor neuron counts, reduced MAP2 immunoreactivity, and a diminished SYN-positive synapse density on ventral motor neurons in SCI mice compared with those in sham-operated controls. In contrast, compared with no treatment and treatment with H-RN, treatment with CAQK-H-RN led to significantly greater preservation of anterior horn neurons, greater MAP2 immunoreactivity, and increased numbers of SYN-positive synapses (Fig. 2D to G).

**Fig. 2.**
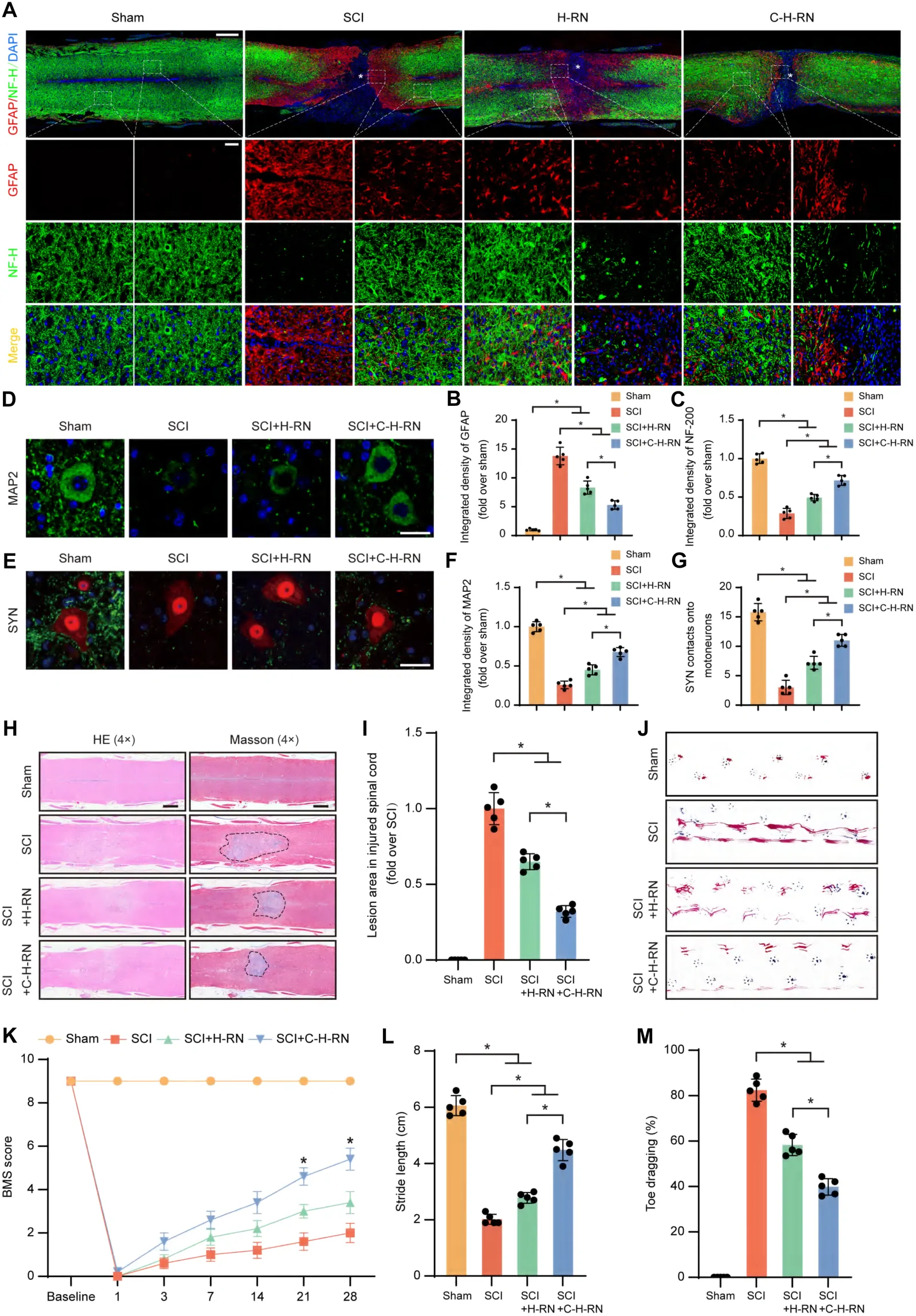
CAQK-H-RN promotes post-spinal cord injury (post-SCI) functional recovery. (A) Representative images of immunofluorescence staining for glial fibrillary acidic protein (GFAP; red) and NF-200 (green) in each group on day 28 after SCI (scale bar = 1,000 μm). Enlarged immunofluorescence images are shown (scale bar = 40 μm). (B and C) Quantitative analysis of the relative integrated density of the fluorescence of glial fibrillary acidic protein (GFAP) and NF-200. (D) Images of the spinal cord cross-sections from the corresponding groups subjected to staining for microtubule-associated protein-2 (MAP2; green) (scale bar = 20 μm). (E) Images of spinal cord cross-sections from the corresponding groups subjected to staining for synaptophysin (SYN; green) and NeuN (red) (scale bar = 20 μm). (F) Quantitative analysis of the relative integrated density of the fluorescence of MAP2. (G) Relevant quantitative results for the number of synapses contacting motor neurons on day 28 after SCI. (H) Longitudinal spinal cord sections from each group at 28 d postinjury (dpi) were examined through hematoxylin–eosin (HE) staining and Masson staining (scale bar = 1,000 μm). (I) Quantitative investigations of Masson-positive lesions within the spinal cords of the respective groups. (J) On day 28 after SCI, images of mouse footprints were captured. Blue, forepaw print; red, hindpaw print. (K) Basso Mouse Scale (BMS) scores were recorded for the groups on days 1, 3, 7, 14, 21, and 28 following SCI. (L and M) Stride length (cm) and toe dragging (%) analyses of the mice at 28 dpi. The data are presented as mean ± standard error of the mean (SEM) (*n* = 5 mice per group); *P < 0.05 indicates significant differences; ns, not significant. For the data in (B), (C), (F), (G), (I), and (K) to (M), statistical analysis was performed using 2-way analysis of variance (ANOVA) followed by Tukey’s multiple comparisons test.

The HE and Masson’s trichrome staining techniques were applied to assess histopathological changes at the injury site. Compared with the sham control group, the SCI group exhibited markedly expanded glial scar formation. The administration of either the H-RN peptide or the synthetic peptide CAQK-H-RN significantly decreased the glial scar area, and CAQK-H-RN treatment resulted in a superior therapeutic effect (Fig. 2H and I). Footprint analysis and BMS scoring were employed to further examine motor functional recovery facilitated by CAQK-H-RN. At 28 dpi, footprint assays revealed significant gait improvement and restored hind-limb mobility in CAQK-H-RN-treated SCI mice, whereas untreated SCI mice were unable to lift their hind limbs (Fig. 2J). The quantitative footprint data, specifically toe dragging and stride length data, corroborated these results (Fig. 2L and M). Moreover, compared with the SCI and H-RN treatments, the CAQK-H-RN treatment significantly increased the BMS at 14, 21, and 28 dpi (Fig. 2K).

Additionally, comprehensive motion tracking and kinematic analyses of hind-limb function were performed using video recordings with the labeling of key hind-limb joints (Fig. 3A). Data from each kinematic parameter are presented in radar charts using 2 characteristic features to provide a concise overview (Fig. 3B). Comparative analysis revealed significant differences between the SCI group and both the sham surgery group and the SCI+H-RN and SCI+C-H-RN treatment groups. Specifically, the SCI group consistently exhibited dragging behavior (Fig. 3C and D). Notably, the SCI group did not present distinctive ankle joint movement features (Fig. 3C). Among the treatment groups, compared with the sham group, only the SCI+C-H-RN group displayed a discernible swing phase, albeit irregular and pathological (Fig. 3C and D). Detailed hind-limb kinematic analysis further revealed marked improvements in the CAQK-H-RN treatment group, including partial restorations of ankle, knee, and hip joint mobility, along with significantly enhanced hind-limb support capability (Fig. 3E to H). These results indicated that CAQK-H-RN treatment effectively promoted functional recovery in SCI model mice.

**Fig. 3.**
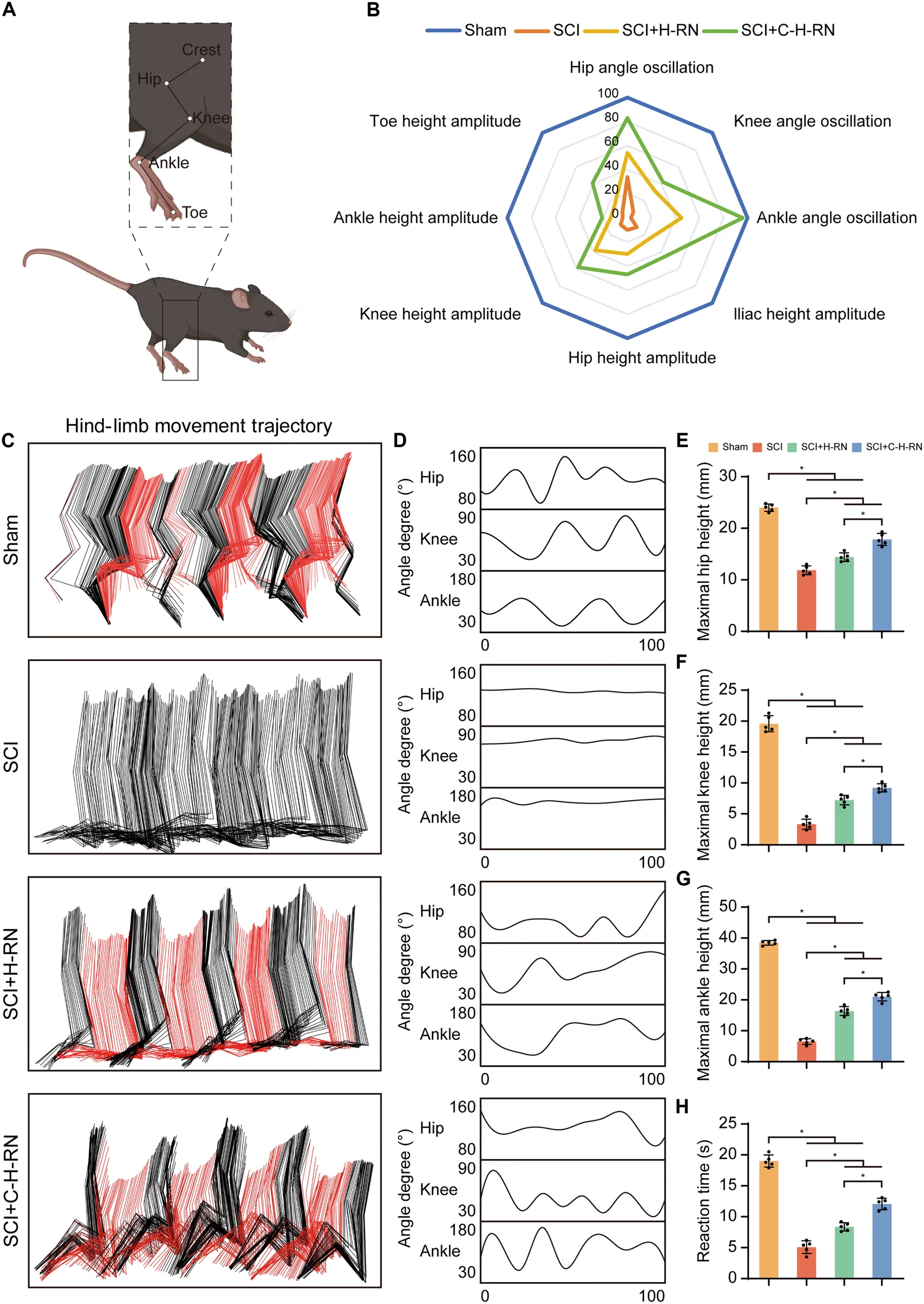
Comprehensive analysis of motor function recovery in mice following CAQK-H-RN therapy. (A) Schematic illustration depicting the coordinated joint movements in the hind limbs of mice during locomotion. (B) Radar chart presenting a quantitative analysis of 8 behavioral parameters in the hind limbs across the 4 experimental groups. (C) Color-coded stick views illustrating hind-limb movements in representative mice from the sham, SCI, SCI+H-RN, and SCI+C-H-RN groups. (D) Angle variation curves depicting hip joint, knee joint, and ankle joint movements during hind-limb locomotion in mice from the sham, SCI, SCI+H-RN, and SCI+C-H-RN groups. (E to H) Quantitative analysis of the maximal hip height, maximal knee height, maximal ankle height, and reaction time in the respective groups. The data are presented as mean ± standard error of the mean (SEM) (*n* = 5 mice per group); *P < 0.05 indicates significant differences; ns, not significant. For the data in (E) to (H), statistical analysis was performed using 2-way analysis of variance (ANOVA) with Tukey’s multiple comparisons test.

### CAQK-H-RN attenuates pyroptosis in neurons after SCI and potentiates neuronal autophagy

Inflammation is an unavoidable outcome following trauma-induced tissue injury. The timely resolution of inflammatory responses is crucial for efficient host defense mechanisms and optimal cellular regeneration; however, excessive or uncontrolled inflammation exacerbates cell death and intensifies tissue injury [28]. Pyroptosis, a type of inflammatory programmed cell death regulated by cysteine-dependent aspartate-specific proteases, has recently garnered considerable attention because of its prominent involvement in inflammatory reactions [28]. We analyzed key molecular components of pyroptotic signaling, including NLRP1, NLRP3, caspase-1, GSDMD-N, IL-1β, IL-18, and ASC, to explore the potential inhibitory effect of the synthetic peptide CAQK-H-RN on pyroptosis. IF staining revealed pronounced neuronal fluorescence signals for caspase-1 and GSDMD-N in the SCI group, whereas significantly diminished signals were observed following CAQK-H-RN administration (SCI+C-H-RN group) (Fig. 4A to C). Additionally, Western blotting (WB) revealed that pyroptosis-related proteins were significantly up-regulated in the SCI group compared with those in the sham group; however, treatment with CAQK-H-RN markedly decreased the levels of all 7 pyroptosis-associated proteins examined (Fig. 4G and H). These findings indicate that CAQK-H-RN effectively suppresses neuronal pyroptosis following SCI. In parallel, excessive reactive oxygen species production is a hallmark of the postinjury microenvironment, acting as both a driver and a consequence of neuroinflammation. Transcriptome sequencing revealed that the oxidative stress response was inhibited in response to CAQK-H-RN peptide treatment after SCI (Fig. 1M). To evaluate the antioxidant capacity of CAQK-H-RN in vivo, we performed DHE staining to measure reactive oxygen species levels in spinal cord tissues on day 3 postinjury (Fig. S3A and B). The SCI group exhibited intense red fluorescence, indicating abundant superoxide generation at the lesion site. In contrast, both the CAQK-H-RN and H-RN treatment groups exhibited decreased integrated DHE fluorescence intensity, with the CAQK-H-RN group showing a lower intensity than the H-RN group, indicating that both treatments reduce oxidative stress but that CAQK-H-RN is more effective.

**Fig. 4.**
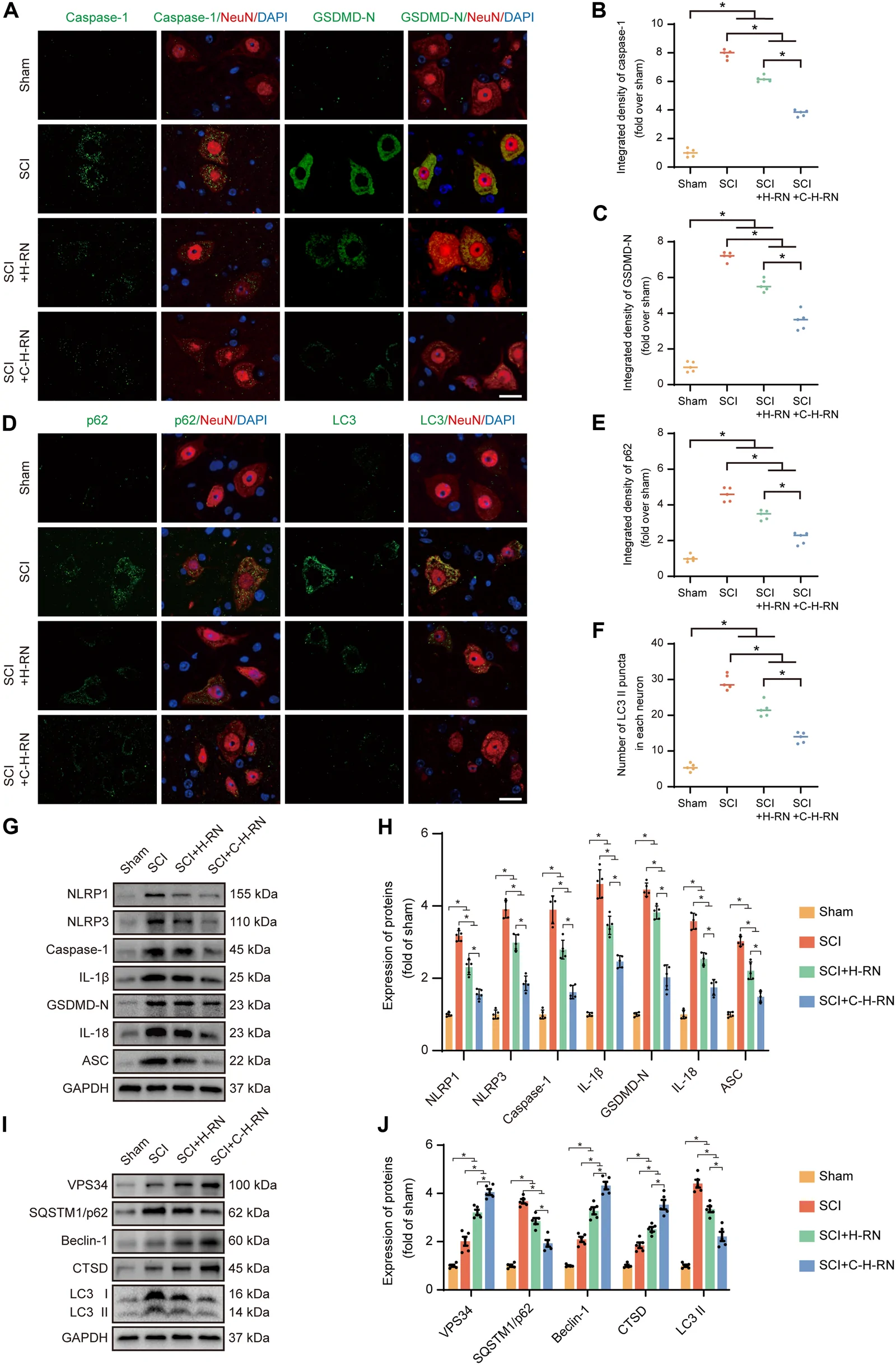
CAQK-H-RN attenuates neuronal pyroptosis after spinal cord injury (SCI) and potentiates neuronal autophagy. (A to C) Double immunofluorescence staining for caspase-1/NeuN and gasdermin D N-terminus (GSDMD-N)/NeuN in the spinal cord ventral horn gray matter from each group (the sham, SCI, SCI+H-RN, and SCI+C-H-RN groups) on day 3 after SCI (scale bar = 20 μm). The graph displays the relative strength of caspase-1 and GSDMD-N expression for each individual neuron. (D to F) Double immunofluorescence staining for p62/NeuN and microtubule-associated protein 1 light chain 3 (LC3)/NeuN in the spinal cord ventral horn gray matter from each group (the sham, SCI, SCI+H-RN, and SCI+C-H-RN groups) on day 3 after SCI (scale bar = 20 μm). The graph displays the integrated density of sequestosome 1 (SQSTM1/p62) and the LC3 II puncta count for each individual neuron. (G) Western blotting (WB) analyses of NLR family pyrin domain containing 1 (NLRP1), NLRP3, caspase-1, interleukin 1β (IL-1β), GSDMD-N, interleukin 18 (IL-18), and apoptosis-associated speck-like protein containing a caspase recruitment segment (ASC) in the injured spinal cord lesion areas. (H) Quantification of the WB results for proteins related to pyroptosis. (I) WB analyses of vacuolar protein sorting 34 (VPS34), SQSTM1, Beclin-1, cathepsin D (CTSD), and LC3 in the injured spinal cord lesion areas. Glyceraldehyde-3-phosphate dehydrogenase (GAPDH) was utilized as a loading control. (J) Quantification of the WB results for proteins related to autophagy. The data are presented as mean ± standard error of the mean (SEM) (*n* = 5 mice per group); *P < 0.05 indicates significant differences; ns, not significant. Statistical analysis was performed using 2-way analysis of variance (ANOVA) followed by Tukey’s multiple comparisons test.

Research has revealed increased autophagy activity following SCI, and increasing evidence has indicated that autophagy acts as a cytoprotective mechanism, promoting neuronal survival by regulating programmed cell death induced by inflammation [29]. We examined, 3 d after injury, the expression of autophagy-related proteins (VPS34, Beclin-1, and LC3), the lysosomal enzyme CTSD, and the autophagy substrate SQSTM1/p62 to evaluate the influence of CAQK-H-RN on neuronal autophagic processes after SCI. The IF results revealed a reduction in the expression of neuronal SQSTM1/p62 in the spinal cord lesions of the mice treated with CAQK-H-RN compared with those of the untreated SCI mice (Fig. 4D and E). Furthermore, impaired autophagy following SCI resulted in increased numbers of LC3 II puncta within neurons; this accumulation was effectively attenuated by both the H-RN peptide and the synthetic peptide CAQK-H-RN, with CAQK-H-RN producing a greater reduction (Fig. 4D and F). WB analyses supported these observations, revealing increased levels of CTSD, VPS34, and Beclin-1 and decreased expression of SQSTM1/p62, LC3 II, and total LC3 in response to CAQK-H-RN treatment (Fig. 4I and J). Together, these data suggest that the synthetic peptide CAQK-H-RN enhances neuronal autophagy by stimulating autophagy–lysosomal pathways following SCI.

### Inhibiting autophagy abrogates CAQK-H-RN-mediated inhibition of neuronal pyroptosis and functional recovery following SCI

CQ, a classical autophagic flux inhibitor that disrupts lysosomal acidification, was used to confirm that enhanced neuronal autophagy mediates the beneficial effects of the synthetic peptide CAQK-H-RN on SCI recovery through the inhibition of neuronal pyroptosis [30]. IF staining revealed increases in neuronal SQSTM1/p62 expression and LC3 II fluorescence intensity in SCI+C-H-RN+CQ mice compared with those in SCI+C-H-RN-treated mice (Fig. 5A to C), indicating that CQ effectively inhibited CAQK-H-RN-induced autophagic flux. WB further revealed no significant changes in Beclin-1 or VPS34 levels between the SCI+C-H-RN and SCI+C-H-RN+CQ groups (Fig. 5G and H), suggesting that autophagosome formation itself was unaffected by CQ in CAQK-H-RN-treated animals. However, CQ markedly increased LC3 II and SQSTM1/p62 expression, which was consistent with the immunostaining data, confirming that CQ blocked CAQK-H-RN-stimulated autophagic degradation (Fig. 5G and H). Autophagy exerts neuroprotective effects by modulating neuronal survival pathways following SCI, whereas pyroptosis constitutes a distinct inflammatory mode of programmed cell death that influences neuronal fate [31]. We assessed the expression levels of pyroptosis-related proteins across groups to determine whether CQ interferes with the ability of CAQK-H-RN to suppress pyroptosis. The IF results revealed increased expression of caspase-1 and GSDMD-N in the SCI+C-H-RN+CQ group compared with that in the SCI+C-H-RN group (Fig. 5D to F). Consistent with the IF findings, WB analysis confirmed that the levels of pyroptosis markers increased in CAQK-H-RN-treated SCI mice following the administration of CQ (Fig. 5I and J). These results suggest that CQ partially abolished the inhibitory effects of CAQK-H-RN on neuronal pyroptosis following SCI.

**Fig. 5.**
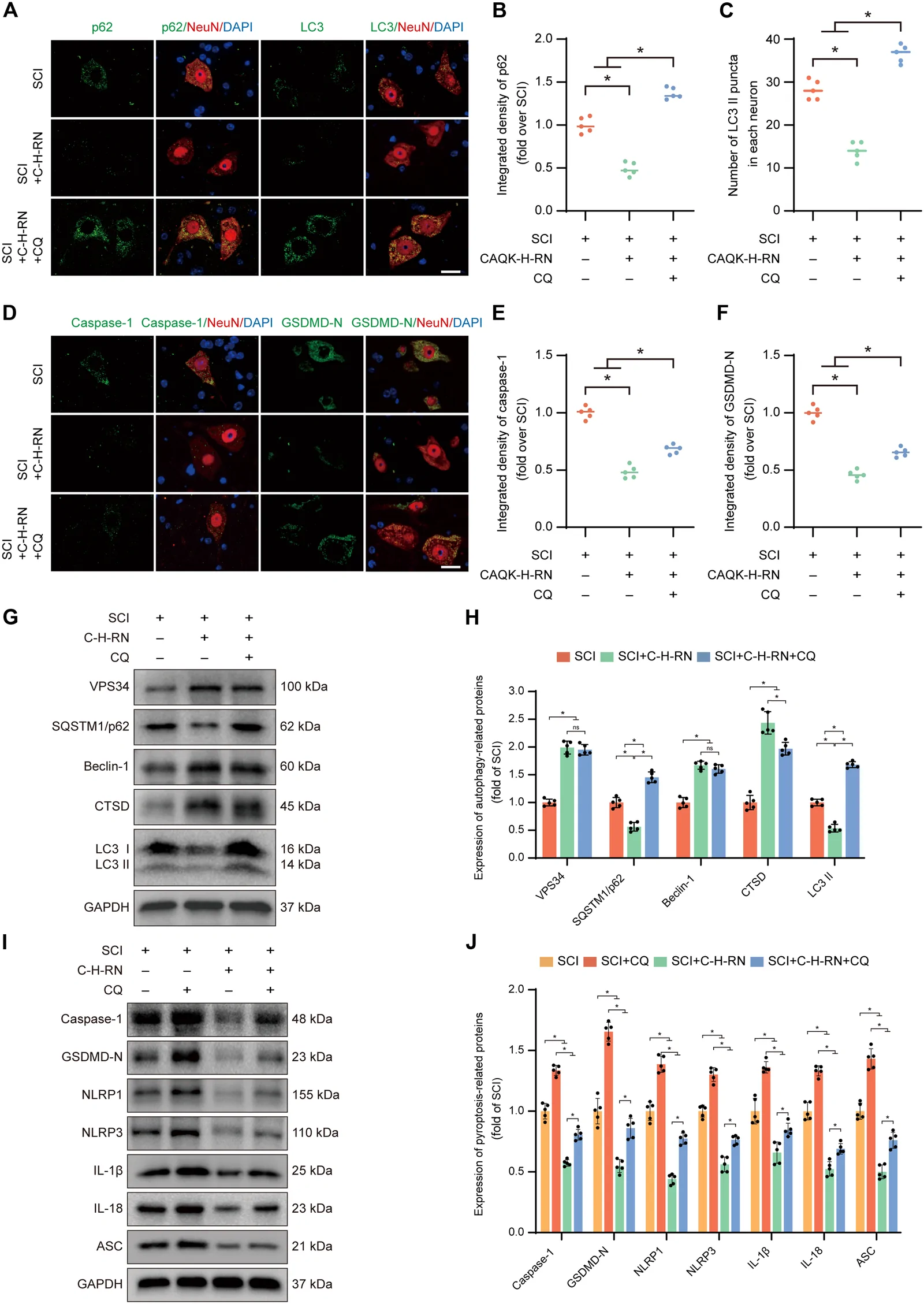
Autophagy inhibition abrogates CAQK-H-RN-mediated neuronal pyroptosis inhibition and functional recovery following spinal cord injury (SCI). (A to C) Double immunofluorescence staining for p62/NeuN and microtubule-associated protein 1 light chain 3 (LC3)/NeuN in the spinal cord ventral horn gray matter from each group (the SCI, SCI+C-H-RN, and SCI+C-H-RN+CQ groups) on day 3 after SCI (scale bar = 20 μm). The graph displays the integrated density of sequestosome 1 (SQSTM1/p62) and the LC3 II puncta count for each individual neuron. (D to F) Double immunofluorescence staining for caspase-1/NeuN and gasdermin D N-terminus (GSDMD-N)/NeuN in the spinal cord ventral horn gray matter from each group (the SCI, SCI+C-H-RN, and SCI+C-H-RN+CQ groups) on day 3 after SCI (scale bar = 20 μm). The graph displays the relative strength of caspase-1 and GSDMD-N expression for each individual neuron. (G) Western blotting (WB) analyses of vacuolar protein sorting 34 (VPS34), SQSTM1/p62, Beclin-1, cathepsin D (CTSD), and LC3 in the injured spinal cord lesion areas. Glyceraldehyde-3-phosphate dehydrogenase (GAPDH) was utilized as a loading control. (H) Quantification of the WB results for proteins related to autophagy. (I) WB analyses of NLR family pyrin domain containing 1 (NLRP1), NLRP3, caspase-1, interleukin 1β (IL-1β), GSDMD-N, interleukin 18 (IL-18), and apoptosis-associated speck-like protein containing a caspase recruitment segment (ASC) in the injured spinal cord lesion areas. (J) Quantification of the WB results for proteins related to pyroptosis. The data are presented as mean ± standard error of the mean (SEM) (*n* = 5 mice per group); *P < 0.05 indicates significant differences; ns, not significant. Statistical analysis was performed using 2-way analysis of variance (ANOVA) followed by Tukey’s multiple comparisons test.

Next, we investigated whether CQ administration compromised the therapeutic efficacy of CAQK-H-RN on motor function recovery after SCI. At 28 dpi, histological assessments (HE and Masson’s trichrome staining), IF staining, and motor performance evaluations were conducted on the SCI, SCI+C-H-RN, and SCI+C-H-RN+CQ groups. IF staining revealed fewer anterior horn motor neurons, diminished NF-H immunoreactivity near the lesion site, increased GFAP expression, reduced MAP2 staining intensity, and fewer SYN-positive synapses on ventral horn motor neurons in SCI+C-H-RN+CQ-treated animals than in SCI+C-H-RN-treated animals (Fig. S4A to G). Moreover, Masson’s trichrome staining revealed an expanded glial scar area at the lesion site in SCI+C-H-RN+CQ-treated mice compared with their SCI+C-H-RN-treated counterparts (Fig. S4H and I). Footprint analysis and quantitative footprint data, specifically toe dragging and stride length data, revealed substantial improvements in hind-limb function and coordination in SCI+C-H-RN mice, whereas SCI+C-H-RN+CQ-treated mice continued to exhibit pronounced hind-limb dragging (Fig. S4J, K, and M). Additionally, significantly lower BMS scores were recorded at 14, 21, and 28 dpi in the SCI+C-H-RN+CQ group than in the SCI+C-H-RN group (Fig. S4L). Collectively, these data highlight the importance of autophagy activation by CAQK-H-RN for its beneficial role in improving motor recovery following SCI.

### CAQK-H-RN attenuates LMP and inhibits the activity of ASMase in neurons after SCI

A previous study reported that LMP, accompanied by the translocation of the lysosomal enzyme CTSB into the cytoplasm, initiates pyroptotic signaling cascades. Accumulating evidence also highlights that impaired lysosomal function disrupts the normal autophagic process, resulting in the accumulation of autophagosomes in neurons in response to neurological conditions, including SCI [32]. Given the notable inhibitory effects of the synthetic peptide CAQK-H-RN on neuronal pyroptosis and autophagic flux, we speculated that CAQK-H-RN might also regulate lysosomal integrity in neurons following SCI. Lysosome-enriched fractions were isolated from spinal cord tissue through subcellular fractionation, and the lysosomal enzyme distribution was assessed using WB and IF staining to investigate this possibility. WB analysis revealed elevated levels of lysosomal enzymes (CTSL, CTSB, and CTSD) within the cytosolic fraction and correspondingly decreased levels in lysosomal fractions in the SCI group compared with those in the sham control group (Fig. 6A to I). Moreover, IF staining for CTSB and the neuronal marker NeuN revealed a diffuse intracellular distribution and decreased punctate staining in neurons from the SCI group compared with those from the sham group (Fig. 6J and K). These findings collectively indicate the significant cytosolic leakage of lysosomal enzymes, providing direct evidence of neuronal LMP following SCI. Notably, the administration of CAQK-H-RN restored the lysosomal enzyme localization, suggesting a protective effect on LMP-induced neuronal injury after SCI (Fig. 6A to K).

**Fig. 6.**
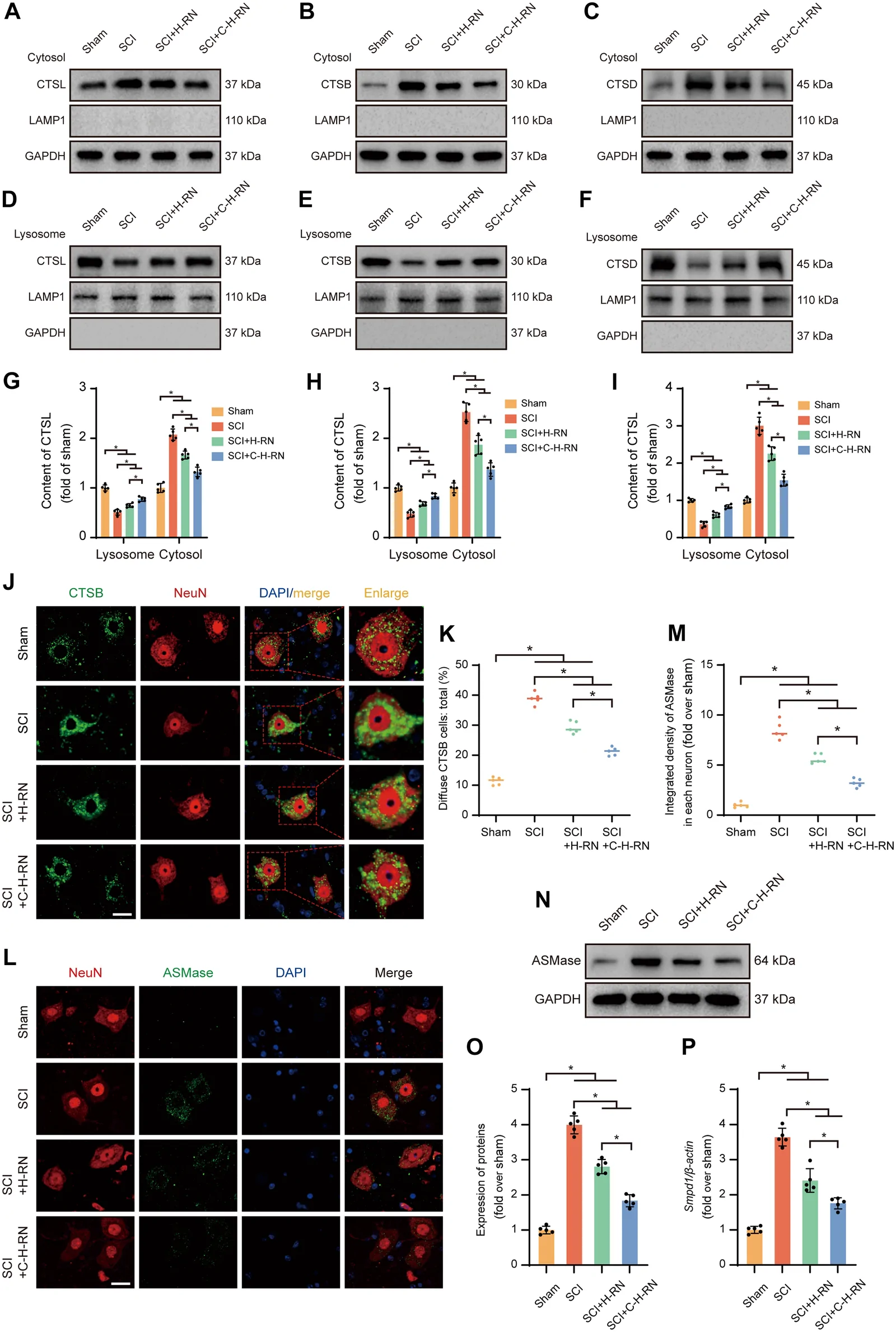
CAQK-H-RN attenuates lysosomal membrane permeabilization (LMP) and inhibits the activity of acid sphingomyelinase (ASMase) in neurons after spinal cord injury (SCI). (A to C) The cytoplasmic protein levels of (A) cathepsin L (CTSL), (B) cathepsin B (CTSB), and (C) cathepsin D (CTSD) were measured in the spinal cords of the sham, SCI, SCI+H-RN, and SCI+C-H-RN groups. Lysosome-associated membrane protein 1 (LAMP1) was utilized as a means of identifying the lysosomal fraction and as a loading control. Glyceraldehyde-3-phosphate dehydrogenase (GAPDH) was used to identify the cytosolic fraction and as a loading control. (D to F) The lysosomal protein levels of (D) CTSL, (E) CTSB, and (F) CTSD were measured in the spinal cords of the sham, SCI, SCI+H-RN, and SCI+C-H-RN groups. LAMP1 was utilized as a means of identifying the lysosomal fraction and as a loading control. GAPDH was used to identify the cytosolic fraction and as a loading control. (G to I) The protein levels of (G) CTSL, (H) CTSB, and (I) CTSD were quantified in both cytoplasmic and lysosomal fractions extracted from the spinal cord. (J) Immunofluorescence (IF) staining was performed to detect the expression of NeuN (red) and CTSB (green) in the anterior horns of the spinal cords from the sham, SCI, SCI+H-RN, and SCI+C-H-RN groups on day 3 after SCI (scale bar = 20 μm). (K) Comparisons were made between the 4 groups regarding the proportions of neurons showing diffuse CTSB localization in the anterior horn of the spinal cord. (L) IF staining was performed to detect the expression of NeuN (red) and ASMase (green) in the anterior horns of the spinal cords from the sham, SCI, SCI+H-RN, and SCI+C-H-RN groups on day 3 after SCI (scale bar = 20 μm). (M) The quantitative integrated density of ASMase in each neuron is shown on the graph. (N) ASMase protein levels in the spinal cords from the 4 groups on day 3 after SCI. (O) The optical density values for ASMase were measured and evaluated in all groups. (P) Messenger RNA (mRNA) levels of the ASMase gene in the injured spinal cord of the groups on day 3 after SCI. The data were normalized to those of β-actin. The data are presented as mean ± standard error of the mean (SEM) (*n* = 5 mice per group); *P < 0.05 indicates significant differences; ns, not significant. Statistical analysis was performed using 2-way analysis of variance (ANOVA) followed by Tukey’s multiple comparisons test.

Taken together, these results suggest that SCI disrupts lysosomal membrane stability, promoting enzyme leakage into the neuronal cytoplasm. Phospholipids are fundamental structural components of neuronal lipid membranes [33]. ASMase converts SM into ceramide [10], which is subsequently hydrolyzed into sphingosine, triggering LMP [34]. Therefore, we hypothesized that the synthetic peptide CAQK-H-RN stabilizes lysosomal membranes after SCI by suppressing ASMase activity. Our earlier research demonstrated peak ASMase activation at 3 d after SCI [12]. Single-cell RNA sequencing datasets derived from the GEO database were analyzed to further clarify ASMase expression patterns across spinal cord cell subtypes following injury. This dataset included transcriptomic profiles across 10 distinct spinal cord cell populations at multiple postinjury time points (Fig. S5A). The results revealed that ASMase is widely distributed across astrocytes, neurons, oligodendrocyte progenitors, and oligodendrocytes (Fig. S5B and C). Furthermore, we found that ASMase expression tended to increase in some cell types at longer durations after SCI (Fig. S5D). Figure S5E shows high ASMase expression on the first day after surgery. The number of ASMase-positive neurons remained high from day 1 through week 3 postsurgery (Fig. S5F). These findings suggest that neurons may be the primary source of ASMase expression and may play a critical role in neuronal pathophysiology. These results highlight the complex temporal regulation of ASMase following SCI, laying the groundwork for further exploration of its neuropathological significance. We evaluated the enzymatic activity of ASMase in neuronal tissues isolated from the sham, SCI, SCI+H-RN, and SCI+C-H-RN groups to determine its role. Our findings revealed a notable increase in ASMase activity after SCI, which was more effectively attenuated by CAQK-H-RN than by the peptide H-RN alone (Fig. 6N to P). Consistent with the IF observations, WB analyses revealed significantly increased ASMase protein expression in the SCI group compared with that in the sham control group, and this increase was notably reversed by CAQK-H-RN administration (Fig. 6L and M). These results suggest that the synthetic peptide CAQK-H-RN effectively inhibits neuronal ASMase activity after SCI. Moreover, the results of the enzyme activity assays corroborated these findings, showing increased ASMase enzymatic activity after injury and its subsequent reduction by CAQK-H-RN treatment (Fig. S6A). Subsequently, lysosome-enriched and cytosolic fractions were isolated from spinal cord tissues for assessment of their enzymatic activity. Lysosomal enzyme assays revealed marked reductions in CTSD and NAGLU enzymatic activities in lysosomal fractions from the SCI group compared with those from the sham-operated group (Fig. S4B and C), alongside significantly elevated enzymatic activity in the corresponding cytosolic fractions (Fig. S6D and E). Notably, compared with treatment with H-RN alone, CAQK-H-RN treatment effectively mitigated these SCI-induced disruptions more robustly, suggesting a restorative effect on lysosomal functionality. Thus, CAQK-H-RN effectively inhibited ASMase phosphorylation, reducing lysosomal membrane impairment in neurons following SCI.

### CAQK-H-RN mitigates LMP, increases autophagy, and suppresses pyroptosis in neurons by inhibiting ASMase after SCI

To further clarify the involvement of ASMase activation in mediating the effects of CAQK-H-RN on LMP, autophagy, and pyroptosis, we employed an AAV vector expressing ASMase to increase ASMase activity in neurons. After AAV vector transduction, obvious enhanced green fluorescent protein fluorescent signals were observed in the neurons, whereas little to no fluorescence was detected in the control group, indicating successful AAV-mediated transduction (Fig. S7A). Five experimental conditions were assessed: SCI, SCI+AAV-Blank, SCI+AAV-ASMase, SCI+C-H-RN+AAV-Blank, and SCI+C-H-RN+AAV-ASMase. IF analyses confirmed significantly increased neuronal ASMase expression in the SCI+C-H-RN+AAV-ASMase group compared with that in the SCI+C-H-RN+AAV-Blank control group (Fig. 7A to E), confirming effective ASMase activation by AAV-mediated gene delivery. WB further revealed increased cytosolic leakage of lysosomal enzymes in the SCI+C-H-RN+AAV-ASMase group compared with that in the SCI+C-H-RN+AAV-Blank group (Fig. 7F to H). Similarly, immunostaining showed an increase in the diffuse cytoplasmic localization of CTSB in neurons from SCI+C-H-RN+AAV-ASMase-treated mice compared with their SCI+C-H-RN+AAV-Blank-treated counterparts (Fig. 7I and J). Enzymatic activity assays further confirmed that compared with those in the SCI and SCI+AAV-Blank groups, the ASMase activity in the SCI+AAV-ASMase group was significantly greater, a trend similar to that noted between the SCI+C-H-RN+AAV-ASMase and SCI+C-H-RN+AAV-Blank groups (Fig. S7B). The ELISA results revealed reduced CTSD and NAGLU enzymatic activities in lysosomal fractions of SCI+AAV-ASMase-treated mice compared with those of SCI+AAV-Blank control mice, and a parallel pattern was observed when the SCI+C-H-RN+AAV-ASMase group was compared with the SCI+C-H-RN+AAV-Blank group (Fig. S7C and D). In contrast, compared with their respective AAV-Blank controls, the cytosolic fractions presented increased CTSD and NAGLU enzymatic activities in both the SCI+AAV-ASMase and SCI+C-H-RN+AAV-ASMase groups (Fig. S7E and F). Collectively, these findings indicate that the synthetic peptide CAQK-H-RN protects against neuronal LMP by inhibiting ASMase activation following SCI.

**Fig. 7.**
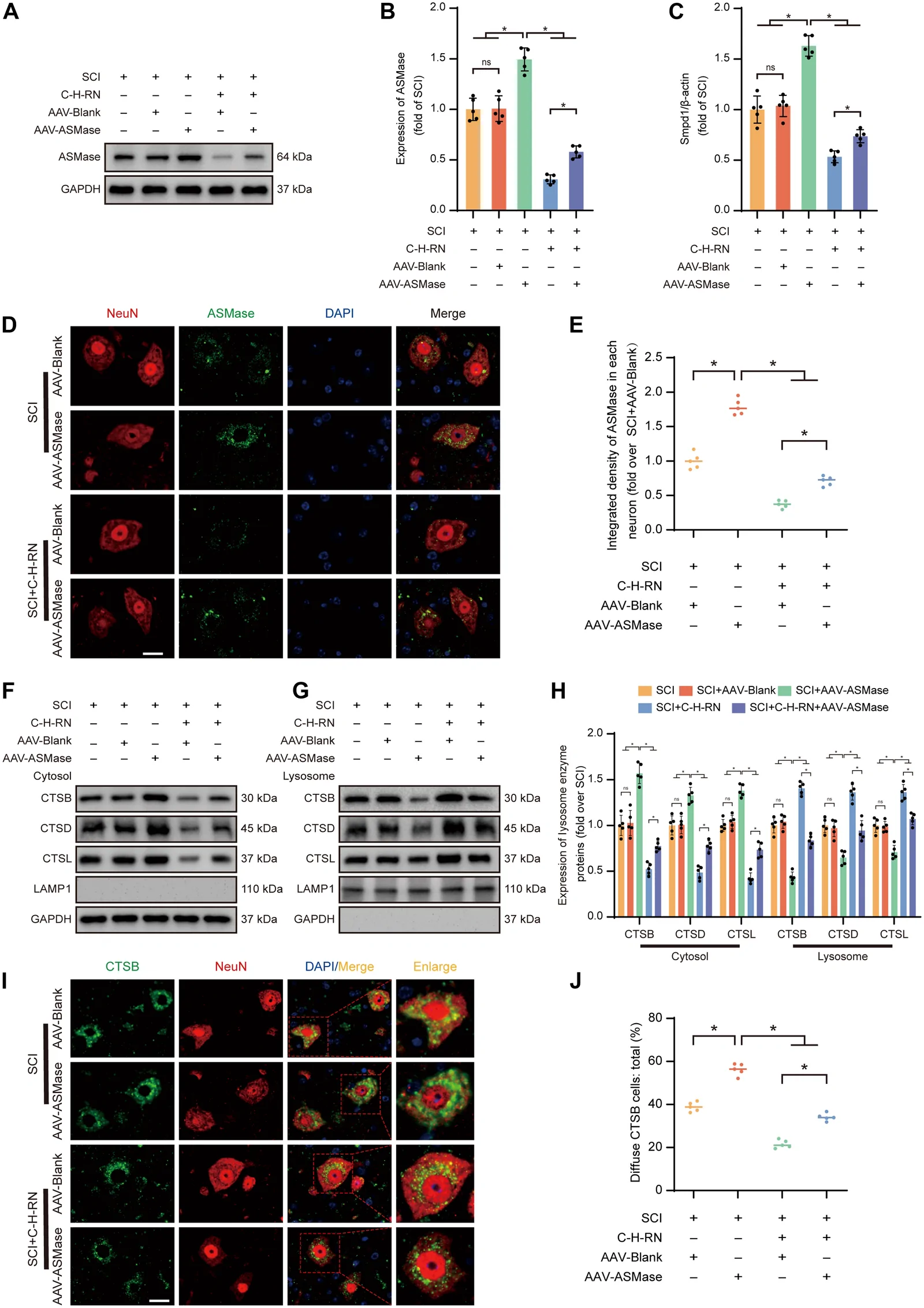
CAQK-H-RN mitigates lysosomal membrane permeabilization (LMP) and increases autophagy in neurons by inhibiting acid sphingomyelinase (ASMase) expression post-spinal cord injury (post-SCI). (A) Western blotting (WB) analysis of ASMase expression levels in the SCI, SCI+AAV-Blank, SCI+AAV-ASMase, SCI+C-H-RN-AAV-Blank, and SCI+C-H-RN-AAV-ASMase groups. Glyceraldehyde-3-phosphate dehydrogenase (GAPDH) was utilized as a loading control. (B) The optical density values for ASMase were measured and evaluated in all groups. (C) Messenger RNA (mRNA) levels of the ASMase gene in the injured spinal cord of the groups on day 3 after SCI. The data were normalized to those of β-actin. (D) Immunofluorescence (IF) staining was performed to detect the expression of NeuN (red) and ASMase (green) in the anterior horns of the spinal cords from the SCI, SCI+AAV-Blank, SCI+AAV-ASMase, SCI+C-H-RN-AAV-Blank, and SCI+C-H-RN-AAV-ASMase groups on day 3 after SCI (scale bar = 20 μm). (E) Graphical representation of the quantitative integrated density of ASMase in each neuron. (F and G) The cytoplasmic and lysosomal protein levels of cathepsin L (CTSL), cathepsin B (CTSB), and cathepsin D (CTSD) were measured in the spinal cords of the SCI, SCI+AAV-Blank, SCI+AAV-ASMase, SCI+C-H-RN-AAV-Blank, and SCI+C-H-RN-AAV-ASMase groups. Lysosome-associated membrane protein 1 (LAMP1) was utilized as a means of identifying the lysosomal fraction and as a loading control. GAPDH was used to identify the cytosolic fraction and as a loading control. (H) The protein levels of CTSB, CTSD, and CTSL were quantified in both cytoplasmic and lysosomal fractions obtained from the spinal cord. (I) IF staining was performed to detect the expression of NeuN (red) and CTSB (green) in the anterior horns of the spinal cords from the SCI, SCI+AAV-Blank, SCI+AAV-ASMase, SCI+C-H-RN-AAV-Blank, and SCI+C-H-RN-AAV-ASMase groups on day 3 after SCI (scale bar = 20 μm). (J) Comparisons were made between the 5 groups regarding the proportions of neurons showing diffuse CTSB localization in the anterior horn of the spinal cord. The data were normalized to those of β-actin. The data are presented as mean ± standard error of the mean (SEM) (*n* = 5 mice per group); *P < 0.05 indicates significant differences; ns, not significant. Statistical analysis was performed using 2-way analysis of variance (ANOVA) followed by Tukey’s multiple comparisons test.

Given the potent inhibitory effects of the synthetic peptide CAQK-H-RN on ASMase activity, we speculated that this peptide could modulate neuronal pyroptosis, oxidative stress, and autophagic processes. DHE staining confirmed the increased integrated fluorescence intensity of DHE in the SCI+AAV-ASMase group compared with that in the SCI+AAV-Blank group. However, the integrated fluorescence intensity of DHE was reduced in the SCI+C-H-RN+AAV-Blank group, whereas this reduction was reversed in the CAQK-H-RN+AAV-ASMase group (Fig. S8A and B). These results indicate that the synthetic peptide CAQK-H-RN inhibits oxidative stress after SCI by suppressing ASMase activity. IF staining revealed that the integrated fluorescence intensities of neuronal GSDMD-N and caspase-1 were significantly greater in the SCI+AAV-ASMase group than in the SCI+AAV-Blank group, whereas these intensities were markedly lower in the SCI+C-H-RN+AAV-Blank group. Notably, the reduction observed in the SCI+C-H-RN+AAV-Blank group was partially reversed in the SCI+C-H-RN+AAV-ASMase group (Fig. S9A to C). WB assays corroborated these findings, revealing decreased levels of pyroptosis-related proteins in the SCI+C-H-RN+AAV-Blank group compared with those in the SCI+AAV-Blank control group, whereas the levels of these proteins were increased in both the SCI+AAV-ASMase and SCI+C-H-RN+AAV-ASMase groups (Fig. S9G and H). Furthermore, IF and WB analyses showed increased neuronal SQSTM1/p62 expression in the SCI+AAV-ASMase group compared with that in the SCI+AAV-Blank group. In contrast, SQSTM1/p62 protein expression was reduced in the SCI+C-H-RN+AAV-Blank group but increased again upon ASMase activation in the SCI+C-H-RN+AAV-ASMase group (Fig. S9D to H). Additionally, LC3 II puncta and protein levels were significantly lower in neurons from SCI+C-H-RN+AAV-Blank mice than in those from SCI+AAV-Blank mice; however, this decrease was reversed in the SCI+C-H-RN+AAV-ASMase group (Fig. S9D to H). In summary, these observations suggest that the synthetic peptide CAQK-H-RN effectively suppresses ASMase activity, thereby mitigating LMP, enhancing neuronal autophagy, and consequently attenuating pyroptosis following SCI. This restoration of autophagic flux further facilitates functional recovery in SCI mice.

Finally, we assessed whether the improved motor function mediated by the synthetic peptide CAQK-H-RN in SCI mice depends on ASMase inhibition via AAV-ASMase transduction. As anticipated, compared with the SCI+C-H-RN+AAV-Blank group, the SCI+C-H-RN+AAV-ASMase group presented a decreased NF-H staining intensity adjacent to the lesion, increased GFAP expression, increased glial scar formation, reduced ventral horn motor neuron numbers, diminished MAP2 expression, and fewer SYN-positive synapses (Fig. S10A to H). Footprint analyses and BMS scoring supported these findings, revealing significantly lower BMS scores at 21 and 28 dpi in SCI+C-H-RN+AAV-ASMase mice than in their SCI+C-H-RN+AAV-Blank counterparts. Furthermore, on day 28 after injury, the hind-limb coordination and mobility of the mice in the SCI+C-H-RN+AAV-Blank group had substantially recovered, whereas the mice in the SCI+C-H-RN+AAV-ASMase group continued dragging their hind limbs (Fig. S10I to L). Collectively, these results strongly support the notion that the therapeutic effects of the synthetic peptide C-H-RN on SCI occur primarily through the inhibition of ASMase activity, thereby reducing neuronal pyroptosis, preventing LMP, and promoting the restoration of autophagic flux to improve functional outcomes.

### CAQK-H-RN inhibits ASMase activity through the p38 MAPK signaling pathway

Transcriptome sequencing revealed the down-regulation of the MAPK signaling cascade in SCI mice following the administration of the synthetic peptide CAQK-H-RN (Fig. 1K). The MAPK pathway predominantly includes 3 branches: ERK, p38, and JNK [12]. Previous studies have shown that the peptide H-RN potently inhibits p38 MAPK activation across diverse disease models [35]. We further elucidated how CAQK-H-RN modulates MAPK signaling in the context of SCI through additional experiments. As depicted in Fig. 8A and B, ASMase, p38, ERK1/2, and JNK were significantly activated following SCI. Administration of CAQK-H-RN notably reduced ASMase expression levels and decreased the ratio of phosphorylated p38 (p-p38/p38), whereas the ratios of phosphorylated ERK1/2 and JNK (p-ERK1/2/ERK1/2 and p-JNK/JNK) remained unchanged between the SCI and SCI+C-H-RN groups. Molecular docking was subsequently employed to assess the binding interactions between CAQK-H-RN and the p38 MAPK protein, with visualizations generated using the PyMOL 2.3.0 software (Fig. 8C). Structural docking revealed stable peptide–protein interactions characterized by hydrogen bonding, hydrophobic interactions, and π–cation interactions between CAQK-H-RN and MAPK11. Collectively, these observations suggest that CAQK-H-RN selectively inhibits the p38 MAPK pathway without affecting ERK or JNK signaling. AA, a known activator of p38 [27], was used to further confirm whether the p38 MAPK pathway mediates the inhibitory effects of CAQK-H-RN on ASMase activity. The results revealed significantly increased p-p38/p38 ratios and increased ASMase expression in the SCI+C-H-RN+AA group compared with those in the SCI+C-H-RN group, confirming effective p38 activation by AA (Fig. 8D to G). WB analyses (Fig. 8E to G) showed higher expression levels of pyroptosis markers (GSDMD-N and caspase-1) and autophagic proteins (SQSTM1/p62 and LC3 II) in the SCI+C-H-RN+AA group than in the SCI+C-H-RN group, underscoring the ability of AA to reverse CAQK-H-RN-induced inhibition of pyroptosis and restoration of autophagy. Moreover, ELISA was utilized to evaluate how AA and CAQK-H-RN influence ASMase activity and LMP. The results revealed a notable increase in ASMase activity in the SCI+C-H-RN+AA group compared with that in the SCI+C-H-RN group (Fig. S11A). Additionally, the lysosomal enzyme activities (CTSD and NAGLU) were significantly lower in the SCI+C-H-RN+AA group than in the SCI+C-H-RN group (Fig. S11B and C). Conversely, compared with those from the SCI+C-H-RN group, the cytosolic fractions from the SCI+C-H-RN+AA group presented markedly increased CTSD and NAGLU enzymatic activities (Fig. S11D and E). These ELISA findings indicate that AA effectively counteracted the protective effects of the synthetic peptide CAQK-H-RN on ASMase activity and lysosomal membrane stability. In summary, the results of the present study indicate that the synthetic peptide CAQK-H-RN inhibits ASMase activation and attenuates LMP in an SCI model by targeting the p38 MAPK/Elk-1/Egr-1 signaling pathway.

**Fig. 8.**
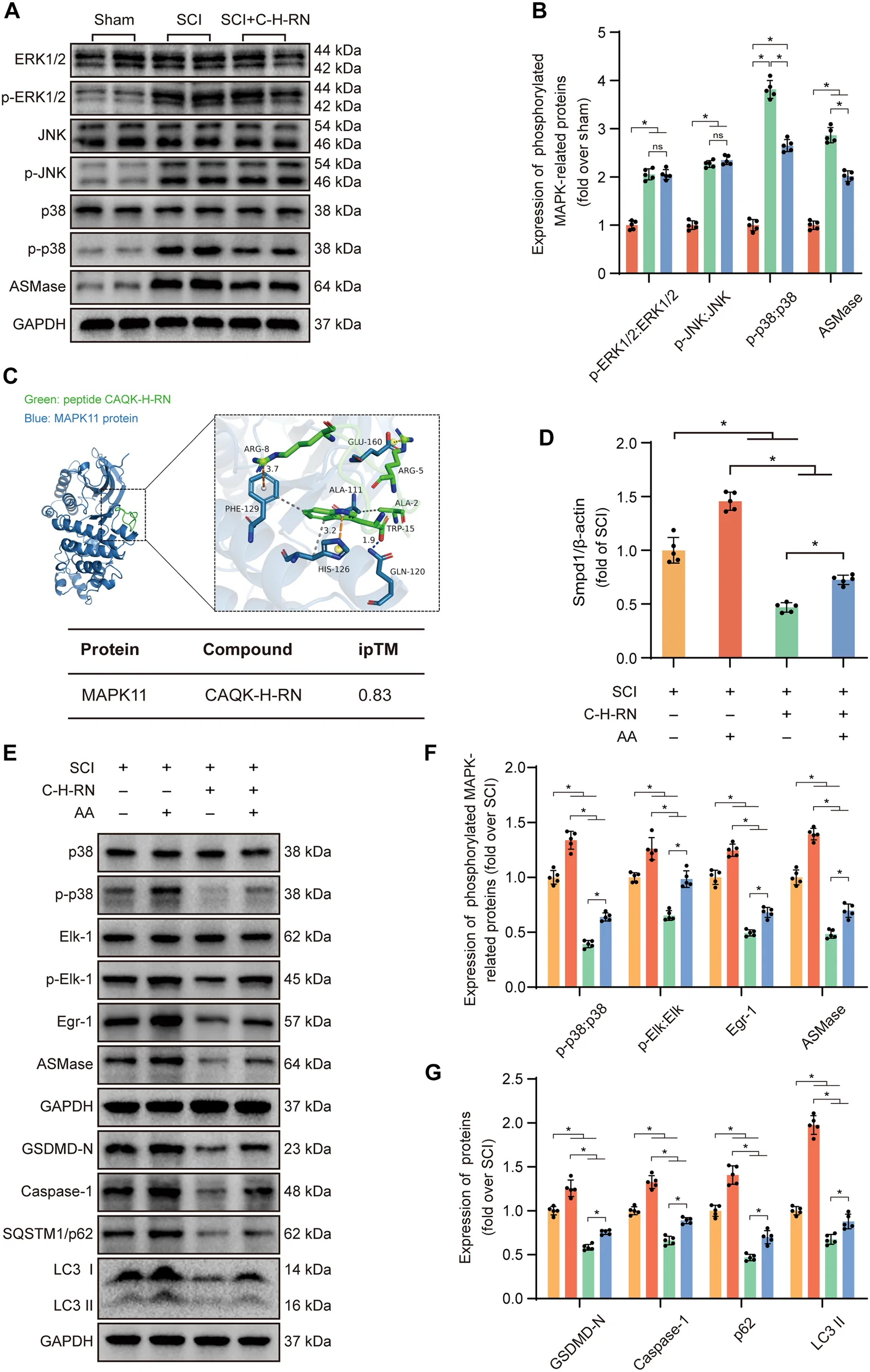
CAQK-H-RN inhibits acid sphingomyelinase (ASMase) activity through the p38 mitogen-activated protein kinase (MAPK)/Ets-like protein-1 (Elk-1)/early growth response protein 1 (Egr-1) signaling pathway. (A) Typical Western blotting (WB) images of extracellular signal-regulated kinase 1/2 (ERK1/2), p-ERK1/2, c-Jun N-terminal kinase (JNK), p-JNK, p38, p-p38, and ASMase expression in the injured spinal cord lesions in the sham, spinal cord injury (SCI), and SCI+C-H-RN groups. Glyceraldehyde-3-phosphate dehydrogenase (GAPDH) was utilized as a loading control. (B) Quantification of the protein levels of ERK1/2, p-ERK1/2, JNK, p-JNK, p38, p-p38, and ASMase. (C) Binding models and 3-dimensional (3D) binding model of the synthetic peptide CAQK-H-RN and MAPK11 complexes. (D) The expression of the ASMase gene in the injured spinal cord on day 3 after SCI was detected using quantitative polymerase chain reaction (qPCR). The data were normalized to those of β-actin. (E) Typical WB images showing p38, p-p38, Elk-1, p-Elk-1, Egr-1, ASMase, GSDMD-N, caspase-1, sequestosome 1 (SQSTM1/p62), and microtubule-associated protein 1 light chain 3 (LC3) II expression in the injured spinal cord lesions in the SCI, SCI+AA, SCI+C-H-RN, and SCI+C-H-RN+AA groups. GAPDH was utilized as a loading control. AA, asiatic acid. (F) Quantification of the protein levels of p38, p-p38, Elk-1, p-Elk-1, Egr-1, and ASMase in the respective groups. (G) Quantification of the protein levels of gasdermin D N-terminus (GSDMD-N), caspase-1, SQSTM1/p62, and LC3 II in the respective groups. The data are presented as mean ± standard error of the mean (SEM) (*n* = 5 mice per group); *P < 0.05 indicates significant differences; ns, not significant. Statistical analysis was performed using 2-way analysis of variance (ANOVA) followed by Tukey’s multiple comparisons test.

## Discussion

Traumatic SCI is a severe neurological disorder that is frequently accompanied by irreversible neuronal impairment, representing a significant therapeutic challenge [1]. Currently, methylprednisolone remains the sole clinical intervention available, primarily providing anti-inflammatory effects in the acute phase. Numerous studies have focused predominantly on secondary injury mechanisms, highlighting neuroinflammation and programmed cell death as central pathological events [3]. Although extensive research efforts have been made to suppress microglial activation and neuronal apoptosis during secondary injury, the therapeutic efficacy of candidate drugs remains limited due to insufficient retention at injury sites, inadequate local accumulation, the absence of controlled release, and poor penetration across the blood–spinal cord barrier [15]. In this study, we synthesized a synthetic peptide, CAQK-H-RN, for the first time, which was administered by intravenous injection to precisely target the site of SCI, improving the efficiency of drug administration and the accumulation of drugs at the site of SCI, thus exerting neuroprotective effects, promoting motor function recovery, and ultimately achieving targeted treatment of SCI. Mechanistically, our GO analysis indicated the significant enrichment of DEGs associated with biological processes involving pyroptosis, autophagy, and MAPK signaling. In this study, SCI induced LMP, disrupted autophagic flux, and activated pyroptotic cell death pathways, ultimately leading to neuronal loss and reduced motor recovery. Importantly, we report that the synthetic peptide CAQK-H-RN mitigated these pathological effects by suppressing the p38–ASMase signaling axis. Therefore, the synthetic peptide CAQK-H-RN represents a promising therapeutic approach for future SCI treatments.

HGF is a versatile endogenous growth factor that is known for its neurotrophic and regenerative properties and exerts beneficial effects on neurological conditions. Previous studies have shown that exogenous HGF delivered through viral vectors substantially enhances neuronal survival, angiogenesis, and axon regeneration, consequently reducing lesion size and promoting motor recovery following SCI [36]. Notably, although classical neurotrophic factors such as brain-derived neurotrophic factor, nerve growth factor, and glial-cell-line-derived neurotrophic factor are essential for neuronal survival and axonal growth, their biological functions are largely confined to neuronal support and synaptic modulation [37]. In contrast, HGF acts as a pleiotropic growth factor that simultaneously targets neurons, glial cells, and vascular endothelial cells through c-Met signaling, thereby exhibiting phase-spanning activity across different stages of SCI, from early cytoprotection and inflammation control to later axonal regeneration and vascular remodeling, whereas the therapeutic window of classical neurotrophic factors is often more constrained [14]. However, the therapeutic application of large biomolecules such as HGF is limited by their short biological half-lives, inefficient targeted delivery, and insufficient penetration of the blood–brain barrier. In this context, small peptides derived from critical functional regions of biologically active proteins have shown considerable potential as therapeutic agents for neurological disorders [38]. Recently, targeted peptide therapy has emerged as an attractive treatment modality across various pathological conditions. Peptides, as natural ligands for multiple cellular receptors, have been extensively employed in targeted drug delivery systems [39]. Owing to their inherent targeting specificity, these peptides preferentially accumulate at injured sites, significantly increasing the efficiency of therapeutic delivery. Additionally, a key property of many small peptides is the ability to permeate the blood–brain barrier, offering a potential additional advantage for CNS targeting [21]. Among these targeting ligands, the CAQK peptide targets SCI, and its targets are CSPGs, which are up-regulated at the injury site. Studies have shown that conjugation with CAQK increases the accumulation of systemically administered molecules, ranging from drug-sized molecules to nanoparticles, at the injury site [40,41]. Although peptides such as the rabies virus glycoprotein are widely used for CNS delivery, they primarily target the ubiquitously expressed nicotinic acetylcholine receptor and thus enable broad CNS distribution but lack injury selectivity [42]. In contrast, CAQK recognizes a pathology-specific signature. CSPGs are spatially and temporally restricted to the glial scar and its periphery, allowing CAQK to achieve lesion-selective accumulation with minimal distribution in uninjured spinal cord regions, thereby reducing off-target toxicity. Moreover, distinct from cell-penetrating peptides such as the trans-activator of transcription peptide, which mediate nonspecific membrane translocation through electrostatic interactions, CAQK relies on specific ligand–receptor binding to the injured extracellular matrix. This mechanism not only enhances local retention but also avoids the indiscriminate cellular uptake associated with cell-penetrating peptides, improving drug delivery efficiency [43]. In addition, persistent extracellular matrix remodeling after SCI provides a stable targeting window for CAQK-mediated delivery beyond the acute phase. Together with its short sequence length, which facilitates synthesis and reduces potential immunogenicity, these features support CAQK as an effective ligand for lesion-specific delivery in SCI. We identified a novel short peptide, H-RN, from the kringle 1 domain of HGF. HGF-derived peptide fragments offer additional advantages over full-length neurotrophic factors. Classical neurotrophic factors possess complex tertiary structures and highly coupled receptor-binding domains, which substantially limit structural modification without compromising biological activity. In contrast, functional motifs derived from HGF, such as kringle-domain-based peptides, provide greater flexibility for molecular engineering, enabling modular integration of targeting, bioactivity, and delivery-related features. Moreover, compared with large synthetic proteins or complex biologics, short-peptide-based designs allow more economical, scalable, and reproducible chemical synthesis and purification, thereby improving translational feasibility. Therefore, we recombined the peptide H-RN with the short peptide CAQK to obtain the synthetic peptide CAQK-H-RN [16]. Currently, no studies have reported the application of H-RN or the synthetic peptide CAQK-H-RN in SCI. Both peptides were administered intravenously to evaluate their therapeutic potential. Compared with H-RN, CAQK-H-RN has superior lesion-targeting ability, resulting in enhanced neuroprotection and improved functional recovery in a murine SCI model. Notably, the canonical biological effects of HGF are mediated through its high-affinity binding to the c-Met receptor, which subsequently activates multiple downstream signaling pathways [44]. Given that H-RN is a peptide fragment derived from HGF, it is necessary to consider whether its biological effects similarly depend on binding to specific cell-surface receptors. Although the classical biological actions of HGF are mediated by c-Met, previous studies have demonstrated that naturally occurring truncated HGF variants, such as NK1, retain partial biological activity without fully recapitulating the receptor activation pattern of full-length HGF [45]. These observations suggest that HGF-derived fragments may exert their effects through noncanonical or modified modes of receptor engagement. However, whether such activities occur entirely independently of c-Met remains to be further elucidated. In the pathological context of SCI, neuronal membrane integrity, receptor distribution, and intracellular signaling responsiveness are profoundly altered. Under such conditions, HGF-derived peptide fragments may exert their biological effects through modified receptor interactions or potentially through receptor-independent mechanisms, including the modulation of intracellular stress signaling and inflammatory cascades. Our current data demonstrate that CAQK-H-RN markedly suppresses inflammasome-associated signaling and neuronal pyroptosis; however, whether these effects are strictly dependent on c-Met binding or involve alternative membrane receptors remains to be determined. Accordingly, we consider this an important mechanistic issue that warrants further investigation.

Pyroptosis has emerged as a key contributor to secondary injury following SCI, as excessive inflammasome activation amplifies neuroinflammation and neuronal loss [4]. Therefore, therapeutic strategies aimed at precisely modulating pyroptosis may be critical for preserving neuronal viability and functional recovery. In the present study, our findings suggest that CAQK-H-RN effectively suppresses neuronal pyroptosis after SCI, highlighting its potential as a neuroprotective intervention. Previous studies from our research group highlighted the involvement of autophagy in SCI pathology [12]. Basal autophagic flux is vital for neuronal survival and cellular homeostasis, and the current evidence suggests that autophagy responses after CNS injuries vary depending on injury location and severity [29]. Increased autophagy following CNS injury reportedly reduces neuronal pyroptosis [12]. Consistent with these findings, we confirmed that pyroptosis levels increased when autophagic flux was impaired after SCI. Importantly, treatment with the synthetic peptide CAQK-H-RN activated neuronal autophagy and markedly inhibited pyroptosis after SCI. Moreover, the beneficial effects of CAQK-H-RN on reducing neuronal pyroptosis and improving functional outcomes were partially reversed when autophagy was inhibited using CQ. Consequently, the synthetic peptide CAQK-H-RN may modulate pyroptosis through a mechanism involving increased autophagy, although additional investigations are necessary to fully clarify this relationship. In summary, our results support that the synthetic peptide CAQK-H-RN exerts therapeutic effects on SCI by promoting autophagic flux and subsequently attenuating neuronal pyroptosis, ultimately facilitating motor recovery. Recently, the contribution of LMP to CNS injuries has become a significant research focus [9]. Notably, current therapeutic strategies targeting LMP in SCI remain inadequate. Based on these findings, we hypothesize that the synthetic peptide CAQK-H-RN may improve neuronal autophagic dysfunction by modulating LMP. Our data substantiate this hypothesis, demonstrating that CAQK-H-RN prevents lysosomal enzyme leakage into the neuronal cytoplasm and restores lysosomal enzyme functionality. Consequently, CAQK-H-RN has therapeutic potential in SCI through the attenuation of LMP and the enhancement of neuronal autophagic flux.

Mechanistically, ASMase plays a central role in regulating lysosomal membrane stability through SM metabolism. Excessive ASMase activation promotes ceramide accumulation, lysosomal destabilization, autophagic disruption, and neuronal death [10]. Our previous work revealed that ASMase is involved in SCI pathology; however, its regulation by CAQK-H-RN has not been explored [12]. The results of the present study provide the first evidence that CAQK-H-RN suppresses neuronal ASMase activation after SCI, thereby alleviating LMP, restoring autophagic flux, and attenuating pyroptosis. These findings identify ASMase as a key molecular target linking CAQK-H-RN to the coordinated regulation of lysosomal function, autophagy, and inflammatory cell death.

We subsequently explored the molecular pathways through which CAQK-H-RN regulates ASMase activity. GO enrichment analysis revealed a significant association between the synthetic peptide CAQK-H-RN and the MAPK signaling cascade. The MAPK family includes 3 primary signaling pathways, ERK, p38, and JNK. Accumulating evidence supports the pivotal role of MAPK signaling in shaping the neuroinflammatory milieu after SCI, with p38, JNK, and ERK acting as key hubs that integrate danger signals and amplify downstream inflammatory cascades. In particular, p38 MAPK has been shown to regulate inflammasome-associated responses and pyroptosis-related signaling in microglia after SCI [46]. For example, Fanconi anemia group C protein (FANCC) overexpression was reported to suppress microglial pyroptosis by inhibiting the p38/NLRP3 axis, thereby mitigating secondary neuronal damage, whereas FANCC knockdown exacerbated these pathological changes [47]. Moreover, modulation of the p38 MAPK pathway via microRNA-based regulation has been reported to relieve SCI-induced secondary neuroinflammation and reduce cell death, further supporting the pathological relevance of p38 activation in SCI [48]. Several previous studies have shown that the peptide H-RN strongly inhibits p38 MAPK activation across various pathological models [35]. Therefore, we hypothesized that CAQK-H-RN exerts its beneficial effects on SCI by targeting the p38 MAPK signaling pathway. Research has shown that SCI activates the p38 MAPK cascade, leading to the nuclear translocation and subsequent phosphorylation of the transcription factor Elk-1. Phosphorylated Elk-1 then forms a transcriptional complex with CREB-binding protein and serum response factor, driving the transcriptional activation of the Egr-1 gene. As a crucial nuclear transcription factor, Egr-1 directly binds the EGR motif in the Smpd1 gene promoter, facilitating ASMase transcription [49]. Our findings show that CAQK-H-RN treatment reduces the activation of both p38 MAPK and ASMase in SCI models. Furthermore, by employing AA, a recognized agonist of p38 MAPK, we confirmed that CAQK-H-RN suppresses ASMase activation via inhibition of the p38 MAPK/Elk-1/Egr-1 signaling axis. Overall, our study provides the first evidence that CAQK-H-RN effectively inhibits ASMase activation through the modulation of the p38 MAPK/Elk-1/Egr-1 pathway in SCI.

The synthetic peptide CAQK-H-RN has demonstrated precise lesion-targeted neuroreparative therapeutic effects in animal models of SCI. Compared with methylprednisolone and neural-stem-cell-based therapies, CAQK-H-RN offers advantages, including lesion specificity, reduced systemic risk, simplified therapeutic procedures, and greater feasibility for manufacturing and regulatory translation, thereby exhibiting substantial translational potential. However, several critical issues remain to be addressed prior to its clinical application. (a) Before clinical trials are initiated, a comprehensive characterization of CAQK-H-RN is needed, including a detailed evaluation of peptide stability, systemic circulation half-life, degradation kinetics, and in vivo biodistribution. (b) While our results demonstrate that the p38 MAPK signaling cascade is selectively inhibited by CAQK-H-RN rather than the ERK1/2 or JNK pathways, the literature suggests that MAPK pathways are interdependently activated following CNS injury [46]. Hence, additional research is essential to clarify whether ERK1/2 or JNK activation could modulate the inhibitory effect of CAQK-H-RN on p38 MAPK signaling. (c) Comparative evaluations of the pharmacokinetic properties of CAQK-H-RN with those of established peptide-based therapeutics or delivery strategies for SCI should be performed in future studies, and CAQK-H-RN should be further integrated with exosome- or nanoparticle-based delivery systems to optimize its pharmacokinetic properties, enhance lesion-site biodistribution, and improve overall translational potential. The modularity of peptide–nanocarrier conjugates offers a flexible platform for iterative optimization. In addition, combination therapy represents a promising avenue for maximizing therapeutic efficacy. CAQK-H-RN could be combined with cell-based strategies targeting neurons or microglia or coadministered with other autophagy-modulating agents to achieve synergistic therapeutic effects and enhanced efficacy. Such multimodal approaches may address the complex pathophysiology of SCI more effectively than single-agent interventions can. (d) Intravenous delivery of CAQK-H-RN offers substantial potential for SCI therapy owing to its targeted localization and high delivery efficiency at lesion sites; however, rigorous clinical trials are necessary to confirm its safety profile and therapeutic efficacy.

This study has certain limitations that warrant further investigation. (a) Although we have demonstrated that impaired lysosomal degradation blocks autophagic flux, the possibility of defective autophagosome–lysosome fusion cannot be excluded. Future studies should extend the treatment duration and include additional treatment time points (e.g., 7, 14, and 56 d) to clearly delineate the dynamic changes in autophagy induced by CAQK-H-RN and to evaluate its long-term effects on motor function recovery after SCI. (b) The HGF/c-Met signaling pathway is a key regulatory axis in liver regeneration. It remains to be determined whether CAQK-H-RN similarly requires binding to specific cell-surface receptors for its downstream biological effects. (c) The pathology of SCI involves complex cellular interactions, with neurons representing the most severely impacted cell type. Neuronal injury contributes substantially to the severity of SCI, underscoring the importance of elucidating neuronal mechanisms to facilitate targeted therapeutic strategies. Notably, our experiments indicate that astrocytes can internalize CAQK-H-RN. Consequently, examining the therapeutic potential of CAQK-H-RN in additional SCI-affected cell types would be beneficial in future studies. (d) Female mice were selected for this study because of the documented difficulties in bladder management after SCI in male mice, as supported by previous investigations. Nonetheless, the potential impact of sex differences on neurological outcomes following SCI must be acknowledged. Previous studies have indicated that estrogen has neuroprotective effects by suppressing inflammatory cascades, mitigating oxidative stress, and preventing neuronal apoptosis, whereas testosterone provides protective benefits to motor neurons and maintains muscular integrity after SCI [50]. However, whether sex hormones influence the therapeutic effectiveness of the synthetic peptide CAQK-H-RN remains uncertain. Hence, evaluating the therapeutic effects of CAQK-H-RN in both male and female SCI mouse models is necessary to obtain a more comprehensive understanding of its therapeutic efficacy.

In summary, our findings provide compelling evidence that the synthetic peptide CAQK-H-RN effectively suppresses neuronal ASMase expression in SCI by specifically targeting injured sites and inhibiting the p38 MAPK/Elk-1/Egr-1 signaling axis. The subsequent reduction in ASMase activity alleviates LMP, restores autophagic flux, and consequently reduces neuronal pyroptosis. These cumulative effects support CAQK-H-RN as a promising candidate for improving the outcomes of SCI patients. The workflow presented in Fig. 9 provides a comprehensive overview of our study. Overall, this research provides novel preclinical insights supporting CAQK-H-RN as a prospective therapeutic intervention for SCI, emphasizing the need for additional research to facilitate its clinical translation.

**Fig. 9.**
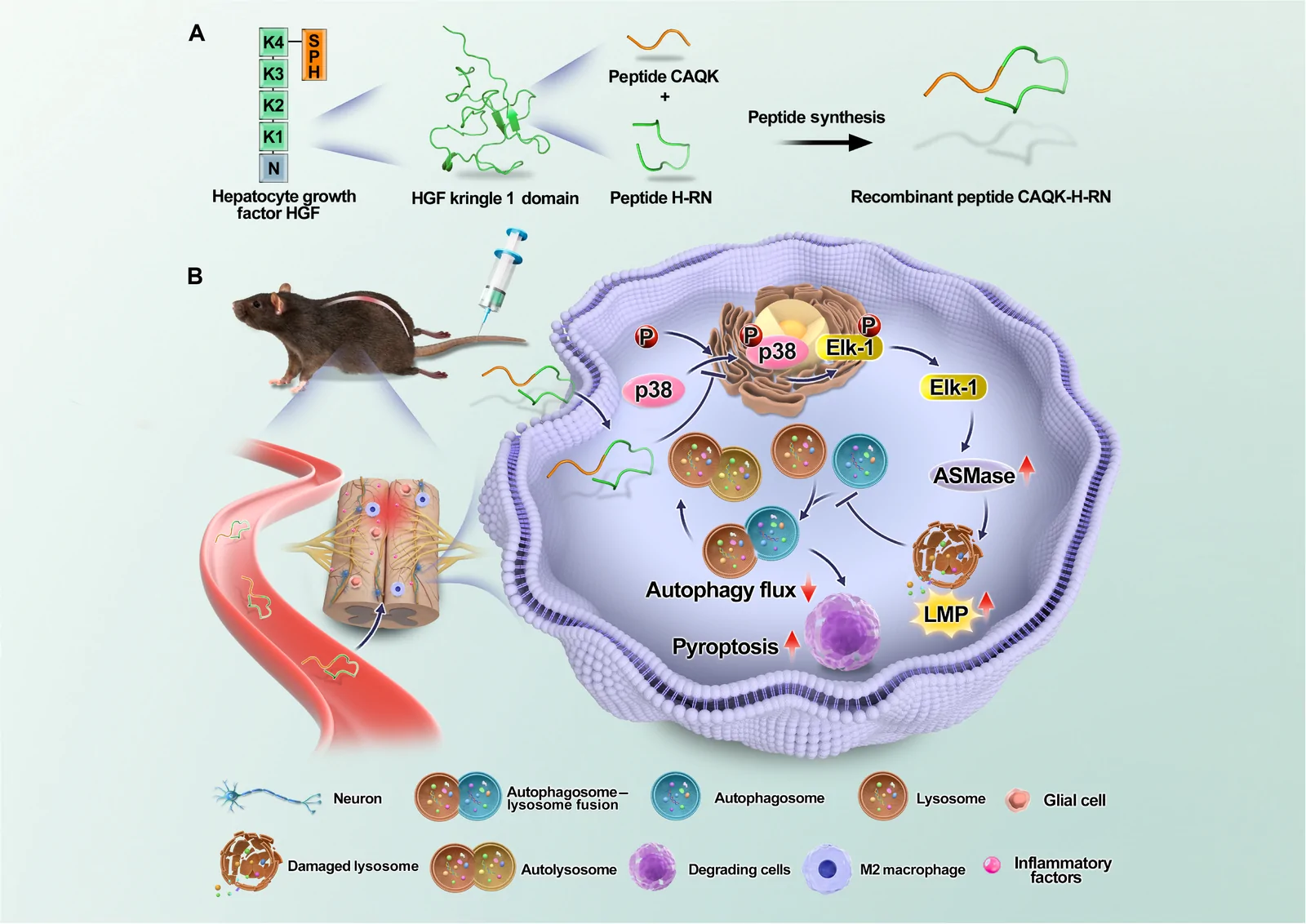
Graphical abstract illustrating the synthesis of the synthetic peptide CAQK-H-RN for targeted delivery to repair spinal cord injury (SCI). (A) Synthesis process of the synthetic peptide CAQK-H-RN. (B) Intravenous injection of the synthetic peptide CAQK-H-RN enables targeted delivery to the SCI site, where it acts on neurons to exert neuroprotective effects and promote axonal regeneration, thereby facilitating the recovery of motor function following SCI.

## Ethical Approval

All animal experiments were conducted in accordance with the ethical standards approved by the ethics committee of Wenzhou Medical University, China (Approval No. wydw2025-0145).

## Data Availability

The data that support the findings of this study are available from the corresponding authors upon reasonable request.

## References

[1] McDonald JW, Sadowsky C. Spinal-cord injury. Lancet. 2002;359(9304):417–425. 10.1016/S0140-6736(02)07603-1 11844532

[2] Ahuja CS, Martin AR, Fehlings M. Recent advances in managing a spinal cord injury secondary to trauma. F1000Res. 2016;5: Article 1017. 10.12688/f1000research.7586.1 PMC4890313 27303644

[3] Mortezaee K, Khanlarkhani N, Beyer C, Zendedel A. Inflammasome: Its role in traumatic brain and spinal cord injury. J Cell Physiol. 2018;233(7):5160–5169. 10.1002/jcp.26287 29150951

[4] Vande Walle L, Lamkanfi M. Pyroptosis. Curr Biol. 2016;26(13):R568–R572. 10.1016/j.cub.2016.02.019 27404251

[5] Zeng C, Wang R, Tan H. Role of pyroptosis in cardiovascular diseases and its therapeutic implications. Int J Biol Sci. 2019;15(7):1345–1357. 10.7150/ijbs.33568 PMC6643148 31337966

[6] Shintani T, Klionsky DJ. Autophagy in health and disease: A double-edged sword. Science. 2004;306(5698):990–995. 10.1126/science.1099993 PMC1705980 15528435

[7] Moujalled D, Strasser A, Liddell JR. Molecular mechanisms of cell death in neurological diseases. Cell Death Differ. 2021;28(7):2029–2044. 10.1038/s41418-021-00814-y PMC8257776 34099897

[8] Gómez-Sintes R, Ledesma MD, Boya P. Lysosomal cell death mechanisms in aging. Ageing Res Rev. 2016;32:150–168. 10.1016/j.arr.2016.02.009 26947122

[9] Li Y, Jones JW, M. C. Choi H, Sarkar C, Kane MA, Koh EY, Lipinski MM, Wu J. cPLA2 activation contributes to lysosomal defects leading to impairment of autophagy after spinal cord injury. Cell Death Dis. 2019;10(7):531. 10.1038/s41419-019-1764-1 PMC6624263 31296844

[10] Henry B, Ziobro R, Becker KA, Kolesnick R, Gulbins E. Acid sphingomyelinase. In: Gulbins E, Petrache I, editors. *Sphingolipids: Basic science and drug development*. Vienna (Austria): Springer; 2013. p. 77–88. 10.1007/978-3-7091-1368-4_4 23579450

[11] Garcia-Ruiz C, Mato JM, Vance D, Kaplowitz N, Fernández-Checa JC. Acid sphingomyelinase-ceramide system in steatohepatitis: A novel target regulating multiple pathways. J Hepatol. 2015;62(1):219–233. 10.1016/j.jhep.2014.09.023 25281863

[12] Geng Y, Lou J, Wu J, Tao Z, Yang N, Kuang J, Wu Y, Zhang J, Xiang L, Shi J, et al. NEMO-binding domain/IKKγ inhibitory peptide alleviates neuronal pyroptosis in spinal cord injury by inhibiting ASMase-induced lysosome membrane permeabilization. Adv Sci. 2024;11(40): Article e2405759. 10.1002/advs.202405759 PMC11516130 39225315

[13] Simonneau C, Bérénice Leclercq BL, Mougel A, Adriaenssens E, Paquet C, Raibaut L, Ollivier N, Drobecq H, Marcoux J, Cianférani S, et al. Semi-synthesis of a HGF/SF kringle one (K1) domain scaffold generates a potent *in vivo* MET receptor agonist. Chem Sci. 2015;6(3):2110–2121. 10.1039/c4sc03856h PMC5496502 28717459

[14] Kitamura K, Nagoshi N, Tsuji O, Matsumoto M, Okano H, Nakamura M. Application of hepatocyte growth factor for acute spinal cord injury: The road from basic studies to human treatment. Int J Mol Sci. 2019;20(5): Article 1054. 10.3390/ijms20051054 PMC6429374 30823442

[15] Hernández-Ledesma B, Hsieh CC, de Lumen BO. Antioxidant and anti-inflammatory properties of cancer preventive peptide lunasin in RAW 264.7 macrophages. Biochem Biophys Res Commun. 2009;390(3):803–808. 10.1016/j.bbrc.2009.10.053 19836349

[16] Wang L, Xu Y, Yu Q, Sun Q, Xu Y, Gu Q, Xu X. H-RN, a novel antiangiogenic peptide derived from hepatocyte growth factor inhibits inflammation in vitro and in vivo through PI3K/AKT/IKK/NF-κB signal pathway. Biochem Pharmacol. 2014;89(2):255–265. 10.1016/j.bcp.2014.02.026 24630926

[17] Zhang H, Chen Y, Li F, Wu C, Cai W, Ye H, Su H, He M, Yang L, Wang X, et al. Elamipretide alleviates pyroptosis in traumatically injured spinal cord by inhibiting cPLA2-induced lysosomal membrane permeabilization. J Neuroinflammation. 2023;20(1):6. 10.1186/s12974-023-02690-4 PMC9825014 36609266

[18] Ge M, Ding Y, Hu T, Chen Y, Shahin V, Li B, Huang T, Qian Y, Zhou Z, Tao Y, et al. Nanomedicine-enabled next-generation therapeutics for spinal cord injury. Mater Today. 2025;86:522–547.

[19] Ding Y, Chen Y, Tao Y, Zhang Z, Ge M, Shi J, Lin H, Xie R. An iron-scavenging and hydrogen-releasing microneedle patch suppresses ferroptosis and promotes spinal cord repair. ACS Nano. 2026;20(16):12210–12226. 10.1021/acsnano.5c19206 41960786

[20] Song YH, Agrawal NK, Griffin JM, Schmidt CE. Recent advances in nanotherapeutic strategies for spinal cord injury repair. Adv Drug Deliv Rev. 2019;148:38–59. 10.1016/j.addr.2018.12.011 PMC6959132 30582938

[21] Wang L, Wang N, Zhang W, Cheng X, Yan Z, Shao G, Wang X, Wang R, Fu C. Therapeutic peptides: Current applications and future directions. Signal Transduct Target Ther. 2022;7(1):48. 10.1038/s41392-022-00904-4 PMC8844085 35165272

[22] Lv Y, Hu Y, Xu Z, Sun H, Lv Y, Dong X, Ye C, Yu G, Dong H, Chang C, et al. An inflammation-targeted lipid nanoparticle inhibiting ferroptosis for spinal cord injury repair. Mater Today Bio. 2026;38: Article 103189. 10.1016/j.mtbio.2026.103189 PMC13156758 42111765

[23] Ge M, Ruan Z, Zhu YX, Wu W, Yang C, Lin H, Shi J. A natural killer cell mimic against intracellular pathogen infections. Sci Adv. 2024;10(44):eadp3976. 10.1126/sciadv.adp3976 PMC11524181 39475620

[24] Qin T, Qin Y, Wen H, Wu T, Duan C, Cao Y, Sun Y, Zhou H, Lu H, Jiang L, et al. Hypoxic neural stem cells enhance spinal cord repair through HIF-1a/RAB17-driven extracellular vesicle release. J Extracell Vesicles. 2025;14(7): Article e70126. 10.1002/jev2.70126 PMC12259395 40660091

[25] Yu L, Zhang Z, Chen H, Wang M, Mao W, Hu J, Zuo D, Lv B, Wu W, Qi S, et al. Remote limb ischemic postconditioning inhibits microglia pyroptosis by modulating HGF after acute ischemia stroke. Bioeng Transl Med. 2023;8(6): Article e10590. 10.1002/btm2.10590 PMC10658568 38023701

[26] Zuo Y, Ye J, Cai W, Guo B, Chen X, Lin L, Jin S, Zheng H, Fang A, Qian X, et al. Controlled delivery of a neurotransmitter–agonist conjugate for functional recovery after severe spinal cord injury. Nat Nanotechnol. 2023;18(10):1230–1240. 10.1038/s41565-023-01416-0 37308588

[27] Wen Z, Mei B, Li H, Dou Y, Tian X, Shen M, Chen G. P2X7 participates in intracerebral hemorrhage-induced secondary brain injury in rats via MAPKs signaling pathways. Neurochem Res. 2017;42(8):2372–2383. 10.1007/s11064-017-2257-1 28488233

[28] Alashmali S, Walchuk C, Cadonic C, Albensi BC, Aliani M, Suh M. The effect of choline availability from gestation to early development on brain and retina functions and phospholipid composition in a male mouse model. Nutr Neurosci. 2022;25(8):1594–1608. 10.1080/1028415X.2021.1885229 33641632

[29] Kassan M, Choi SK, Galán M, Trebak M, Belmadani S, Matrougui K. Nuclear factor kappa B inhibition improves conductance artery function in type 2 diabetic mice. Diabetes Metab Res Rev. 2015;31(1):39–49. 10.1002/dmrr.2542 PMC4829069 24652705

[30] Pan D, Li Y, Yang F, Lv Z, Zhu S, Shao Y, Huang Y, Ning G, Feng S. Increasing toll-like receptor 2 on astrocytes induced by Schwann cell-derived exosomes promotes recovery by inhibiting CSPGs deposition after spinal cord injury. J Neuroinflammation. 2021;18(1):172. 10.1186/s12974-021-02215-x PMC8353762 34372877

[31] Fu H, Zhao Y, Hu D, Wang S, Yu T, Zhang L. Depletion of microglia exacerbates injury and impairs function recovery after spinal cord injury in mice. Cell Death Dis. 2020;11(7):528. 10.1038/s41419-020-2733-4 PMC7359318 32661227

[32] Byers JS, Huguenard AL, Kuruppu D, Liu NK, Xu XM, Sengelaub DR. Neuroprotective effects of testosterone on motoneuron and muscle morphology following spinal cord injury. J Comp Neurol. 2012;520(12):2683–2696. 10.1002/cne.23066 PMC3960947 22314886

[33] Muri J, Kopf M. Redox regulation of immunometabolism. Nat Rev Immunol. 2021;21(6):363–381. 10.1038/s41577-020-00478-8 33340021

[34] Morad SA, Cabot MC. Ceramide-orchestrated signalling in cancer cells. Nat Rev Cancer. 2013;13(1):51–65. 10.1038/nrc3398 23235911

[35] Zhu S, Xu X, Wang L, Su L, Gu Q, Wei F, Liu K. Inhibitory effect of a novel peptide, H-RN, on keratitis induced by LPS or poly(I:C) in vitro and in vivo via suppressing NF-κB and MAPK activation. J Transl Med. 2017;15(1):20. 10.1186/s12967-017-1121-z PMC5270222 28125988

[36] Date I, Takagi N, Takagi K, Kago T, Matsumoto K, Nakamura T, Takeo S. Hepatocyte growth factor improved learning and memory dysfunction of microsphere-embolized rats. J Neurosci Res. 2004;78(3):442–453. 10.1002/jnr.20263 15389834

[37] Gordon T. The role of neurotrophic factors in nerve regeneration. Neurosurg Focus. 2009;26(2):E3. 10.3171/FOC.2009.26.2.E3 19228105

[38] Sims SK, Wilken-Resman B, Smith CJ, Mitchell A, McGonegal L, Sims-Robinson C. Brain-derived neurotrophic factor and nerve growth factor therapeutics for brain injury: The current translational challenges in preclinical and clinical research. Neural Plast. 2022;2022(1):3889300. 10.1155/2022/3889300 PMC8906958 35283994

[39] Kong X, Liu H, Chen S, Liu Z, Chen Q, Li X, Hu H, Su J, Shi Y. Bioengineered bacterial extracellular vesicles for targeted delivery of an osteoclastogenesis-inhibitory peptide to alleviate osteoporosis. J Control Release. 2025;382: Article 113751. 10.1016/j.jconrel.2025.113751 40268198

[40] Castillo JA Jr, Le MN, Ratcliff A, Soufi K, Huang K, Vatoofy S, Ghaffari-Rafi A, Emerson S, Reynolds E, Pivetti C, et al. Systematic review of peptide CAQK: Properties, applications, and outcomes. Int J Mol Sci. 2024;25(20): Article 10990. 10.3390/ijms252010990 PMC11507173 39456774

[41] Ge M, Guo Z, Ruan Z, Ma Y, Zhang Z, Dong H, Shi T, Hu T, Lu L, Chen Y, et al. Chemical substrate-enabled piezoelectric metabolic reprogramming therapy unlocks the vicious triad of diabetic wounds. Cell Biomater. 2026;2(6): Article 100276.

[42] Ding L, Xiao Y, Xu Z, Zhu Z, Gan T, Huang X, Shu H, Liang X, Mo M, Huang X, et al. RVG-targeted extracellular vesicles loaded with echinatin attenuate dopaminergic neurodegeneration via the IGF-2/PI3K/Akt pathway in Parkinson’s disease mice. J Nanobiotechnology. 2026;24(1): Article 117. 10.1186/s12951-025-03997-5 PMC12870896 41495703

[43] Zhou H, Yan L, Huang H, Li X, Xia Q, Zheng L, Shao B, Gao Q, Sun N, Shi J. Tat-NTS peptide protects neurons against cerebral ischemia-reperfusion injury via ANXA1 SUMOylation in microglia. Theranostics. 2023;13(15):5561–5583. 10.7150/thno.85390 PMC10614677 37908731

[44] Uchikawa E, Chen Z, Xiao GY, Zhang X, Bai XC. Structural basis of the activation of c-MET receptor. Nat Commun. 2021;12(1):4074. 10.1038/s41467-021-24367-3 PMC8249616 34210960

[45] Tahira Y, Sakai K, Sato H, Imamura R, Matsumoto K. Dimer interface in natural variant NK1 is dispensable for HGF-dependent Met receptor activation. Int J Mol Sci. 2021;22(17): Article 9240. 10.3390/ijms22179240 PMC8431453 34502141

[46] Jiang F, Xia M, Zhang Y, Chang J, Cao J, Zhang Z, Qian Z, Yang L. Cannabinoid receptor-2 attenuates neuroinflammation by promoting autophagy-mediated degradation of the NLRP3 inflammasome post spinal cord injury. Front Immunol. 2022;13: Article 993168. 10.3389/fimmu.2022.993168 PMC9553321 36238284

[47] Xia M, Li X, Ye S, Zhang Q, Zhao T, Li R, Zhang Y, Xian M, Li T, Li H, et al. FANCC deficiency mediates microglial pyroptosis and secondary neuronal apoptosis in spinal cord contusion. Cell Biosci. 2022;12(1):82. 10.1186/s13578-022-00816-4 PMC9164466 35659106

[48] Qian Z, Chang J, Jiang F, Ge D, Yang L, Li Y, Chen H, Cao X. Excess administration of miR-340-5p ameliorates spinal cord injury-induced neuroinflammation and apoptosis by modulating the P38-MAPK signaling pathway. Brain Behav Immun. 2020;87:531–542. 10.1016/j.bbi.2020.01.025 32014577

[49] Hu J, Han H, Cao P, Yu W, Yang C, Gao Y, Yuan W. Resveratrol improves neuron protection and functional recovery through enhancement of autophagy after spinal cord injury in mice. Am J Transl Res. 2017;9(10):4607–4616. PMC5666068 29118921

[50] Pang QM, Chen SY, Xu QJ, Fu SP, Yang YC, Zou WH, Zhang M, Liu J, Wan WH, Peng JC, et al. Neuroinflammation and scarring after spinal cord injury: Therapeutic roles of MSCs on inflammation and glial scar. Front Immunol. 2021;12: Article 751021. 10.3389/fimmu.2021.751021 PMC8674561 34925326

