# Supplementary material for: Glial Scar-Targeted Modification of Hepatocyte Growth Factor-Derived Peptide for Spinal Cord Injury Therapeutic Repair
Source: Biomater Res. 2026 Oct 27;30:0431. doi: 10.34133/bmr.0431 (PMC13710766; doi:10.34133/bmr.0431)
Supplement: Supplementary file 1 [file bmr.0431.f1.docx]

**Supplementary Information**

**For**

Glial scar-targeted modification of hepatocyte growth factor-derived peptide for spinal cord injury therapeutic repair

Yuzhe Wu et. al

**Abbreviations**

AAV=Adeno-associated virus

ASC=Apoptosis-associated speck-like protein containing a caspase recruitment segment

ASMase=Acid sphingomyelinase

BMS=Basso Mouse Scale

Cer=Ceramide

C-H-RN= synthetic peptide CAQK-H-RN

CNS=Central nervous system

CPPs=cell-penetrating peptides

CQ=Chloroquine

CSPG=Chondroitin sulfate proteoglycan

CTSB=Cathepsin B

CTSD=Cathepsin D

CTSL=Cathepsin L

**Cy5.5=Cyanine5.5**

DAPI=4′,6-diamidino-2-phenylindole

DHE=dihydroethidium

Egr-1=Early growth response-1

Elk-1=Ets-like protein-1

GFAP=Glial fibrillary acidic protein

GO=Gene Ontology

GSDMD=Gasdermin D

HE=Haematoxylin-eosin

HGF=Hepatocyte growth factor

IL-1β=Interleukin 1β

IL-18=Interleukin 18

IF=**Immunofluorescence**

IVIS=**In Vivo Imaging System**

LC3=Microtubule-associated protein 1 light chain 3

MAPK=Mitogen-activated protein kinase

MAP2=Microtubule-associated protein-2

NAGLU=alpha-N-acetylglucosamini-dase

NF-H=Neurofilament heavy polypepide

NLRP1=NLR family pyrin domain containing 1

NLRP3=NLR family pyrin domain containing 3

NLRs=NOD-like receptors

PMA=Phorbol 12-myristate 13-acetate

qPCR=quantitative PCR

ROS=reactive oxygen species

SCI=Spinal cord injury

SM=Sphingomyelin

Sph=Sphingosine

SPH=Serine protease homology domain

SYN=Synaptophysin

TLRs=Toll-like receptors

WB=Western blotting

**Figures and figure legends**

Figure S1

**Figure S1 Biosafety of synthetic peptide CAQK-H-RN and the relationship between the dose and the founctional recovery after SCI with the peptide treatment.** (A) Dose-response chart showing the optimal dosage of synthetic peptide CAQK-H-RN (300ug/kg) for 28 days after SCI for evaluation of BMS score. (B) Bodyweight of mice after intravenous injection of CAQK-H-RN on days 0, 1, 3 and 7. (C-D) Liver function test results 1 day after CAQK-H-RN injection. (C) Alanine aminotransferase. (D) Aspartate aminotransferase. (E) HE staining of major organs 1 day after CAQK-H-RN injection. (scale bar = 50 μm) (F) Kidney function test results 1 day after CAQK-H-RN injection. Creatinine clearance rate. The data are presented as the means ± SEMs (n = 5 mice per group); **p* < 0.05, indicate significant differences; ns, not significant. Statistical analysis was performed using two-way ANOVA followed by Tukey’s multiple comparison test.

Figure S2

**Figure S2 synthetic peptide C-H-RN upregulates in neurons of spinal cord injury area.** (A) Representative immunofluorescence images showing colocalization of Cy5.5 (red) with GFAP, Iba-1 or NeuN (green) in the injured area of mice 3 days after SCI. Nucleus were visualized with DAPI (blue). (scale bar = 40 μm) (B-D) Intensity profiles of indicated proteins along the plotted lines, as analyzed by Image J line scan analysis. (E) Pearson’s correlation coefficient between Cy5.5 intensity and the intensity of NeuN, GFAP and Iba-1. (n = 5 mice per group).

Figure S3

**Figure S3 synthetic peptide C-H-RN inhibits oxidative stress after SCI.** (A) On day 3 after SCI, spinal cord tissue sections from four groups were stained with DHE (scale bar = 20 μm). (B) Quantification of DHE staining in the four groups. The data are presented as the means ± SEMs (n = 5 mice per group); *P < 0.05, indicate significant differences; ns, not significant. Statistical analysis was performed using two-way ANOVA followed by Tukey’s multiple comparison test.

Figure S4

**Figure S4 Inhibiting autophagy with CQ abrogates CAQK-H-RN-mediated functional recovery following SCI.** (A) Representative images of immunofluorescence staining of GFAP (red) and NF-200 (green) in the SCI, SCI+C-H-RN and SCI+C-H-RN+CQ groups on day 28 after SCI. (scale bar = 1000 μm). The enlarged immunofluorescence images form. (scale bar = 40 μm). (B-C) Quantitative analysis of the relative intergrated density of the fluorescence of GFAP and NF-200. (D) Images of spinal cord cross-sections from the corresponding groups were subjected to staining with MAP2 (green). (scale bar = 20 μm). (E) Images of spinal cord cross-sections from the corresponding groups were subjected to staining with SYN (green) and NeuN (red). (scale bar = 20 μm). (F) Quantitative analysis of the relative intergrated density of the fluorescence of MAP2. (G) Relevant quantitative results for motor neuron-contacting synapse amounts on day 28 after SCI. (H) The longitudinal spinal cord sections of each group at 28 dpi were examined through H&E staining and Masson staining (scale bar = 1000 μm). (I) Quantitative investigations of Masson-positive lesions within the spinal cords of the respective groups. (J) Stride length (cm) analyses of mice at 28 dpi. (K) On day 28 after SCI, images of mouse footprints were captured. Blue: fore paw print; Red: hind paw print. (L) The groups' BMS scores were recorded on days 1, 3, 7, 14, 21, and 28 following SCI. (M) Toe dragging (%) analyses of mice at 28 dpi. The data are presented as the means ± SEMs (n = 5 mice per group); **p* < 0.05, indicate significant differences; ns, not significant. Statistical analysis was performed using two-way ANOVA followed by Tukey’s multiple comparison test.

Figure S5

**Figure S5 Overall distribution of *Smpd1* in different cells after SCI.** (A) Uniform Manifold Approximation and Projection (UMAP) plot showing the 10 major cell types of spinal cords. Each dot corresponds to a single cell, colored according to cell type. (B) UMAP projection of the overall distribution of *Smpd1*. (C) Scatter and Violin plots showing the expression distribution of *Smpd1* post injury. (D) UMAP projection of the uninjured, 1d, 1w, 3w and 6w. (E) Scatter and Violin plots showing the expression distribution of *Smpd1* at different time points post injury. (F) The bar chart showing the statistical number of cells expressing *Smpd1* gene in different cell types at different times.

Figure S6

**Figure S6 CAQK-H-RN attenuates LMP and inhibits the activity of ASMase after SCI.** (A) ELISA results indicating the level of ASMase activity in the spinal cord of the Sham, SCI, SCI+H-RN and SCI+C-H-RN groups on day 3 after SCI. (B-E) ELISA was used to determine the activity of the lysosomal enzymes CTSD and NAGLU in both the lysosomal (B, C) and cytosolic (D, E) fractions obtained from the spinal cords of the Sham, SCI, SCI+H-RN and SCI+C-H-RN groups on day 3 postsurgery. The data are presented as the means ± SEMs (n = 5 mice per group); *P < 0.05 indicates significant differences; ns, not significant. Statistical analysis was performed using two-way ANOVA followed by Tukey’s multiple comparison test.

Figure S7

**Figure S7 CAQK-H-RN attenuates LMP by inhibiting ASMase in SCI model mice.** (A) Representative fluorescence image of adenovirus-mediated EGFP expression. (scale bar = 40 μm). (B) ELISA results indicating the level of ASMase activity in the spinal cord of the SCI+AAV-Blank, SCI+AAV-ASMase, SCI+C-H-RN +AAV-Blank and SCI+C-H-RN+AAV-ASMase groups on the third day after surgery. (B-E) ELISA was used to determine the activity of the lysosomal enzymes CTSD and NAGLU in both the lysosomal (C, D) and cytosolic (E, F) fractions obtained from the spinal cords of the SCI+AAV-Blank, SCI+AAV-ASMase, SCI+C-H-RN +AAV-Blank, and SCI+C-H-RN+AAV-ASMase groups on day 3 postsurgery. The data are presented as the means ± SEMs (n = 5 mice per group); *P < 0.05 indicates significant differences; ns, not significant. Statistical analysis was performed using two-way ANOVA followed by Tukey’s multiple comparison test.

Figure S8

**Figure S8 CAQK-H-RN inhibits oxidative stress after SCI by suppressing ASMase activity.** (A) On day 3 after SCI, spinal cord tissue sections from four groups were stained with DHE (scale bar = 20 μm). (B) Quantification of DHE staining in the four groups. The data are presented as the means ± SEMs (n = 5 mice per group); *P < 0.05, indicate significant differences; ns, not significant. Statistical analysis was performed using two-way ANOVA followed by Tukey’s multiple comparison test.

Figure S9

**Figure S9 CAQK-H-RN inhibits pyroptosis and enhances autophagy in neurons by downregulating ASMase.** (A-C) Double immunofluorescence staining for Caspase-1/NeuN and GSDMD-N/NeuN in the spinal cord ventral horn grey matter from each group (the SCI, SCI+AAV-Blank, SCI+AAV-ASMase, SCI+C-H-RN-AAV-Blank and SCI+C-H-RN-AAV-ASMase groups) on day 3 after SCI. (scale bar = 20 μm). The graph displays the relative strength of Caspase-1 and GSDMD-N for each individual neuron. (D-F) Double immunofluorescence staining for p62/NeuN and LC3/NeuN in the spinal cord ventral horn grey matter from each group (the SCI, SCI+AAV-Blank, SCI+AAV-ASMase, SCI+C-H-RN-AAV-Blank and SCI+C-H-RN-AAV-ASMase groups) on day 3 after SCI. (scale bar = 20 μm). The graph displays the the integrated density of SQSTM1/p62 and LC3 II puncta count for each individual neuron. (G) WB analyses of NLRP1, NLRP3, Caspase-1, GSDMD-N, IL-1β, IL-18, ASC, SQSTM1/p62 and LC3 II in the injured spinal cord lesion areas. GAPDH was utilized as a loading control. (H) Quantification results of the related protein levels in the respective groups. The data are presented as the means ± SEMs (n = 5 mice per group); **p* < 0.05, indicate significant differences; ns, not significant. Statistical analysis was performed using two-way ANOVA followed by Tukey’s multiple comparison test.

Figure S10

**Figure S10 CAQK-H-RN facilitates functional recovery in SCI mice by inhibiting ASMase activity.** (A) Representative images of immunofluorescence staining of GFAP (red) and NF-200 (green) in the SCI+AAV-Blank, SCI+AAV-ASMase, SCI+C-H-RN+AAV-Blank and SCI+C-H-RN+AAV-ASMase groups on day 28 after SCI. (scale bar = 1000 μm). The enlarged immunofluorescence images form. (scale bar = 40 μm). (B-C) Quantitative analysis of the relative intergrated density of the fluorescence of GFAP and NF-200. (D) Images of spinal cord cross-sections from the corresponding groups were subjected to staining with MAP2 (green). (scale bar = 20 μm). Images of spinal cord cross-sections from the corresponding groups were subjected to staining with SYN (green) and NeuN (red). (scale bar = 20 μm). (E) Quantitative analysis of the relative intergrated density of the fluorescence of MAP2. (F) Relevant quantitative results for motor neuron-contacting synapse amounts on day 28 after SCI. (G) The longitudinal spinal cord sections of each group at 28 dpi were examined through H&E staining and Masson staining (scale bar = 1000 μm). (H) Quantitative investigations of Masson-positive lesions within the spinal cords of the respective groups. (I) Stride length (cm) analyses of mice at 28 dpi. (J) On day 28 after SCI, images of mouse footprints were captured. Blue: fore paw print; Red: hind paw print. (K) The groups' BMS scores were recorded on days 1, 3, 7, 14, 21, and 28 following SCI. (L) Toe dragging (%) analyses of mice at 28 dpi. The data are presented as the means ± SEMs (n = 5 mice per group); **p* < 0.05, indicate significant differences; ns, not significant. Statistical analysis was performed using two-way ANOVA followed by Tukey’s multiple comparison test.

Figure S11

**Figure S11 CAQK-H-RN attenuates LMP by inhibiting the p38-MAPK/ElK-1/Egr-1 signaling pathway.** (A) ELISA results indicating the level of ASMase activity in the spinal cord of the SCI, SCI+AA, SCI+C-H-RN and SCI+C-H-RN+AA groups on the third day after surgery. (B-E) ELISA was used to determine the activity of the lysosomal enzymes CTSD and NAGLU in both the lysosomal (B, C) and cytosolic (D, E) fractions obtained from the spinal cords of the SCI, SCI+AA, SCI+C-H-RN and SCI+C-H-RN+AA groups on day 3 postsurgery. The data are presented as the means ± SEMs (n = 5 mice per group); *P < 0.05 indicates significant differences; ns, not significant. Statistical analysis was performed using two-way ANOVA followed by Tukey’s multiple comparison test.

**Western Blot Source Date**

**Uncropped original Western Blot images for Figure 4**

Groups: Sham; SCI; SCI+H-RN; SCI+C-H-RN

NLRP1

NLRP3

Caspase-1

IL-1β

GSDMD-N

IL-18

ASC

GAPDH

Groups: Sham; SCI; SCI+H-RN; SCI+C-H-RN

VPS34

P62

Beclin-1

CTSD

LC3

GAPDH

**Uncropped original Western Blot images for Figure 5**

Groups: SCI; SCI+C-H-RN; SCI+C-H-RN+CQ

VPS34

p62

Beclin-1

CTSD

LC3

GAPDH

Groups: SCI; SCI+CQ; SCI+C-H-RN; SCI+C-H-RN+CQ

Caspase-1

GSDMD-N

NLRP3

NLRP1

IL-1β

IL-18

ASC

GAPDH

**Uncropped original Western Blot images for Figure 6**

Groups: Sham; SCI; SCI+H-RN; SCI+C-H-RN

CTSL

LAMP-1

GAPDH

CTSB

LAMP-1

GAPDH

CTSD

LAMP-1

GAPDH

CTSL

LAMP-1

GAPDH

CTSB

LAMP-1

GAPDH

CTSD

LAMP-1

GAPDH

ASMase

GAPDH

**Uncropped original Western Blot images for Figure 7**

Groups: SCI; SCI+AAV-Blank; SCI+AAV-ASMase; SCI+C-H-RN+AAV-Blank; SCI+C-H-RN+AAV-ASMase

ASMase

GAPDH

Groups: SCI; SCI+AAV-Blank; SCI+AAV-ASMase; SCI+C-H-RN+AAV-Blank; SCI+C-H-RN+AAV-ASMase

CTSB

CTSD

CTSL

LAMP-1

GAPDH

CTSB

CTSD

CTSL

LAMP-1

GAPDH

**Uncropped original Western Blot images for Figure 8**

Groups: Sham; SCI; SCI+C-H-RN

ERK1/2

p-ERK1/2

JNK

p-JNK

p38

p-p38

ASMase

GAPDH

Groups: SCI; SCI+AA; SCI+C-H-RN; SCI+C-H-RN+AA

p38

p-p38

Elk-1

P-Elk-1

Egr-1

ASMase

GAPDH

Groups: SCI; SCI+AA; SCI+C-H-RN; SCI+C-H-RN+AA

GSDMD-N

Caspase-1

p62

LC3

GAPDH

**Uncropped original Western Blot images for Figure S9**

Groups: SCI; SCI+AAV-Blank; SCI+AAV-ASMase; SCI+C-H-RN+AAV-Blank; SCI+C-H-RN+AAV-ASMase

NLRP1

NLRP3

Caspase-1

GDMD-N

IL-1β

IL-18

ASC

p62

LC3

GAPDH
